# Denitrogenative Alkylation of K‑Ras(G12D) Inhibits Oncogenic Signaling in Cancer Cells

**DOI:** 10.1021/jacs.5c06745

**Published:** 2025-06-30

**Authors:** Qinheng Zheng, Kevan M. Shokat

**Affiliations:** † Department of Cellular and Molecular Pharmacology and Howard Hughes Medical Institute, 8785 University of California, San Francisco, California 94158, United States; ‡ Department of Chemistry, University of California, Berkeley, California 94720, United States

## Abstract

Pancreatic ductal adenocarcinoma (PDAC) is the most lethal common cancer. More than 90% of PDAC tumors are caused by *KRAS* mutations, with the majority expressing the K-Ras­(G12D) oncoprotein. Despite extensive drug discovery efforts across academia and industry, there are no approved drugs directly targeting K-Ras­(G12D) in a mutant-selective manner. We report a series of α-diazoacetamide compounds that form covalent bonds via denitrogenative alkylation of acquired aspartic acid at the mutation site. The lead molecule allosterically inhibits the mitogen-activated protein kinase pathway downstream of K-Ras and therefore inhibits the growth of *KRAS* ^G12D^-driven cancer cell lines but not non-G12D mutation cancer cell lines. Our results show that the diazo-carboxy ligation spares not only the unreactive Gly12 residue in the K-Ras wild-type protein but also strong nucleophiles such as the Cys12 residue in K-Ras­(G12C). The preference for a weak nucleophile carboxylic acid over canonically stronger nucleophiles provides the basis to expand the covalently targetable proteome to aspartic and glutamic acids.

## Graphical abstract

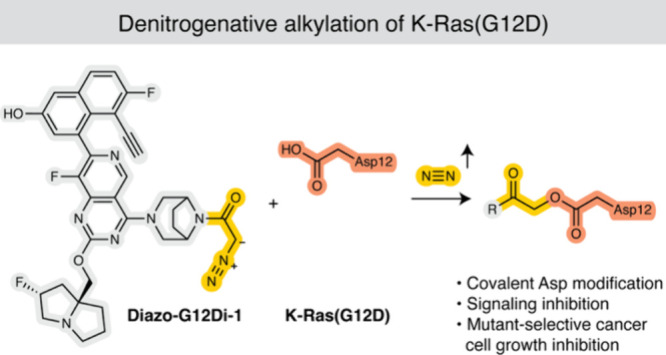

## Introduction

*KRAS* is the most frequently mutated human oncogene, accounting for 30% of all human cancers.^1^ It has taken three decades for researchers to find the first molecules to bind K-Ras, against one particular mutant G12C that has a chemically tractable acquired Cys12.^2^ These covalent allosteric inhibitors cross-link Cys12 and bind to a cryptic pocket now known as the Switch-II Pocket (S-IIP). The discovery of S-IIP has sparked the resurgence of K-Ras drug discovery, leading to two US FDA-approved drugs, sotorasib and adagrasib, to treat K-Ras­(G12C) mutation driven non-small-cell lung cancer.^3,4^ However, there are no approved drugs targeting the most common K-Ras mutation, G12D.

The K-Ras­(G12D) mutation occurs in 27% of pancreatic, 10% of colon, and 4% of lung adenocarcinoma patients.^5–7^ New chemical matter has emerged to explore the noncovalent engagement with the S-IIP^8^ or the neo-protein–protein interface between Ras and cyclophilin A.^9,10^ These compounds with K-Ras­(G12D), pan-K-Ras, or pan-Ras selectivity have entered clinical trials and demonstrated promising results. Meanwhile, we reasoned that targeted covalent inhibitors^11,12^ may benefit from enhanced potency, mutant selectivity, and more durable pharmacodynamics to the existing noncovalent chemotypes. Furthermore, since chemotypes for covalent binding to Asp and Glu are rare, understanding how to covalently modify these weakly nucleophilic amino acids may enable targeting of other targets.

We hypothesize that one can redirect K-Ras­(G12C) covalent inhibitors to target G12D with carboxylic-acid-specific covalent chemistry. Recent reports exploited the ring strain of heterocyclic groups, such as β-lactones,^13^ epoxides,^14,15^ and aziridines,^16,17^ to cross-link Asp12. These covalent G12D inhibitors are dual G12C/G12D inhibitors. Their reactivity with Cys12 introduces potential liabilities such as inactivation by small molecule thiols in complex biological systems. A chemoselective functional group, in contrast, offers an improved balance between reactivity and stability. Here we report the discovery of α-diazoacetamides as targeted covalent K-Ras­(G12D) inhibitors with improved efficacy and exclusive G12D mutant selectivity.

The reactivity toward acid is a characteristic property of diazo compounds^18^ since their discovery by Curtius in 1883.^19^ Staudinger and Gaule later demonstrated^20^ the dependence of the stability of diazo compounds on their basicity. An α-carbonyl substituent dramatically stabilizes diazo groups by lowering their p*K*_a_ and contributing to a charge separated resonance structure. The stability of the α-diazo carbonyl moiety is exemplified by their presence in natural products azaserine^21,22^ and 6-diazo-5-oxo-*L*-norleucine^23^ (DON). Like its 1,3-dipolar isostere azide, diazo compounds are emerging as useful tools for chemical biology as summarized by Raines et al. in 2016.^24^ The most prominent applications are the bioreversible modification of protein surface aspartic and glutamic acids^25–28^ and analysis of carboxylic acid metabolites.^29–31^ Both exploit the chemoselectivity for carboxylic acids in a biological milieu.

Meanwhile, the use of diazo compounds as targeted covalent inhibitors to treat cancer remains elusive. A report from our lab first showed the potential to use a diazo grafted S-IIP ligand to covalently modify the acquired Asp12 of K-Ras­(G12D).^17^ Similar results were observed by Li and co-workers in a recent report.^14^ Both compounds require high concentrations (mM) to achieve appreciable covalent modification in biochemical assays with unknown cellular activity.

## Results and Discussion

Our recently reported cocrystal structure of a malolactone-cross-linked K-Ras­(G12D) suggested a stable ester linkage between the S-IIP ligand MRTX1133 ^8,32^ and K-Ras­(G12D). We focused on the backbone of the ester linkage and designed an α-diazoacetamide (**Diazo-G12Di-1**) by warhead hopping and retrosynthetic analysis via a denitrogenative alkylation pathway (Figure 1A,B and Figure S1). The proposed covalent linker, obtained from a covalent docking protocol,^33^ between **Diazo-G12Di-1** and Asp12 aligned with that in our previously reported crystal structure (Figure S2).

**Figure 1:**
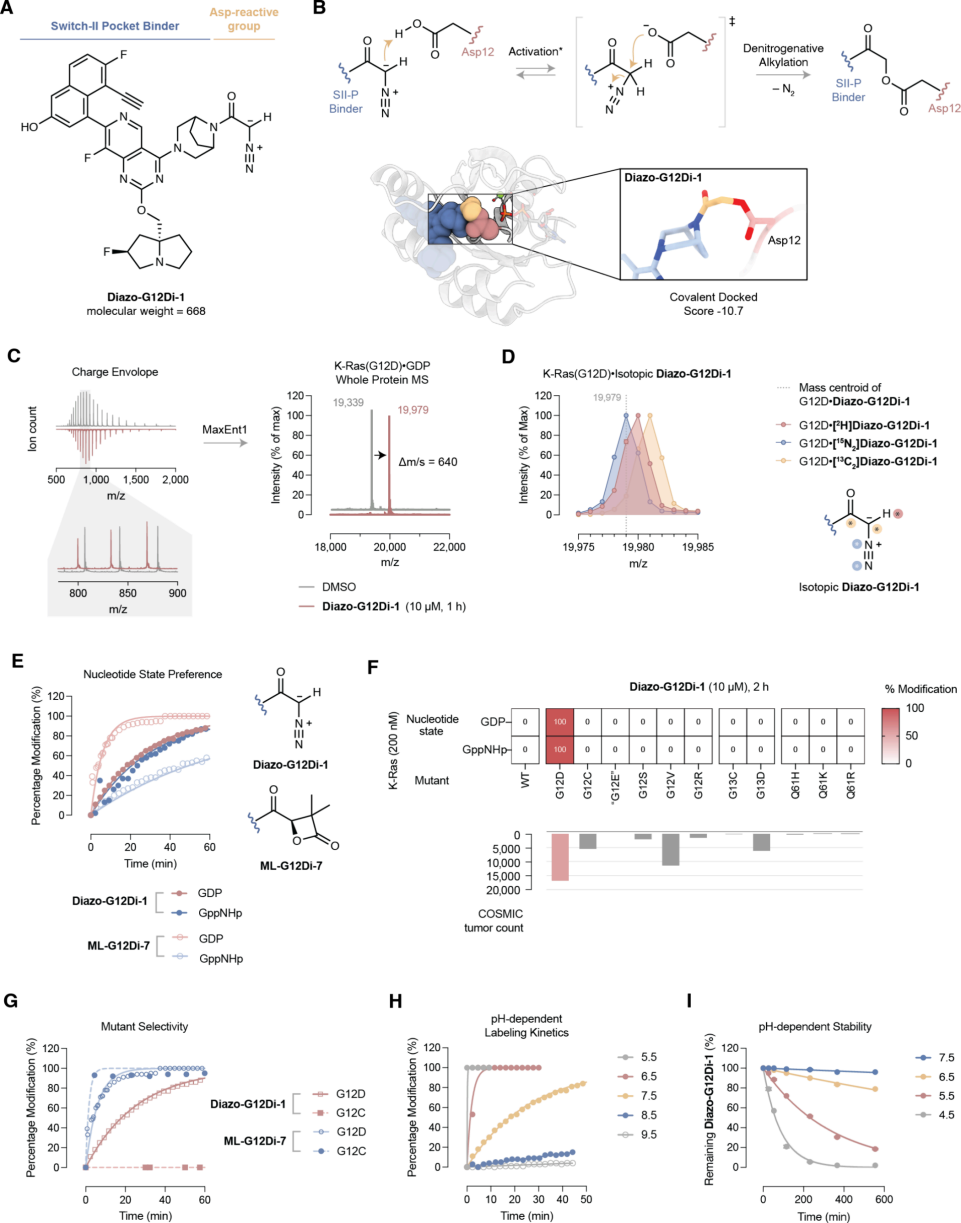
(A) Chemical structure of **Diazo-G12Di-1**. (B) Denitrogenative alkylation of Asp12 by **Diazo-G12Di-1**. *Proton transfer could occur from either aspartic acid or bulk solvent through hydrogen bonding networks. **Diazo-G12Di-1** was covalently docked into K-Ras­(G12D)•GDP (PDB 7RPZ) using a customized carboxylic acid alkylation reaction type. (C) Protein charge envelops and deconvoluted intact protein MS. Conditions: 200 nM K-Ras­(G12D) incubated without (gray) or with (salmon) 10 μM **Diazo-G12Di-1** at 20 °C for 1 h. (D) Intact protein MS of K-Ras­(G12D) modified by isotopically labeled derivatives of **Diazo-G12Di-1**. (E) Comparative covalent modification kinetics for GDP- and GppNHp-bound K-Ras­(G12D) with **Diazo-G12Di-1** or **ML-G12Di-7**. (F) Covalent modification selectivity of **Diazo-G12Di-1** against cancer-related K-Ras mutants and their occurrence in cancer. (G) Comparison of G12C/D selectivity between **Diazo-G12Di-1** and **ML-G12Di-7**. (H) pH-dependent covalent modification kinetics. (I) pH-dependent chemical stability of **Diazo-G12Di-1.**.

**Diazo-G12Di-1** reacted with purified recombinant K-Ras­(G12D) protein, resulting in a mass shifted species in intact protein mass spectrometry (MS); the gain in mass corresponded to the mass of **Diazo-G12Di-1**’s molecular weight subtracted by 28 mass units (Figure 1C). To probe the mechanism of the covalent modification, we prepared three isotopically labeled compounds, i.e., [^2^H]**Diazo-G12Di-1**, [^15^N_2_]**Diazo-G12Di-1**, and [^13^C_2_]**Diazo-G12Di-1**. [^15^N_2_]**Diazo-G12Di-1** led to no appreciable difference in the high-resolution Q-TOF MS, while [^2^H]**Diazo-G12Di-1** and [^13^C_2_]**Diazo-G12Di-1** resulted in further mass gain (Figure 1D). Therefore, we reasoned that the 28 mass unit offset was due to the loss of a dinitrogen in the covalent modification process via a well-known denitrogenative alkylation mechanism. The proposed mechanism is depicted in Figure 1B. The α-diazoacetamide is brought to the proximity of Asp12, where it deprotonates Asp12 to form an ion pair. The activated diazonium then alkylates the carboxylate of Asp12, yielding the stable ligand–protein adduct.

Oncogenic mutations of K-Ras disrupt its GTPase activity.^34^ K-Ras oncoproteins in cancer cells are enriched in their GTP-bound signaling competent state, or “on”-state. Approved K-Ras­(G12C) drugs trap the GDP-state, or “off”-state, to indirectly target the “on”-state protein.^35^ **Diazo-G12Di-1** covalently engaged with both GDP- and GTP-bound purified K-Ras­(G12D) proteins. The covalent modification of recombinant K-Ras­(G12D) protein was efficient, achieving full labeling in about 1 h (Figure 1E). The “on”-state targeting potentially confers faster signaling pathway suppression in cancer cells.^13^

We further tested the chemoselectivity of the carboxy-diazo ligation. We selected a panel of K-Ras proteins including wild-type and cancer-causing hotspot mutations. While the unbound structures of these K-Ras proteins are highly superimposable with max root-mean-square deviation^36^ (r.m.s.d.) values of 1.01 Å (Table S1), **Diazo-G12Di-1** demonstrated excellent selectivity for K-Ras­(G12D) in both “on”- and “off”-states (Figure 1F). Wild-type K-Ras was not modified in the MS-based covalent engagement assay. Analogous mutants to G12D, including G13D and G12E, were not modified by **Diazo-G12Di-1**, indicating the stringent geometry requirement for the occurrence of Asp/Glu ligation on top of proximity. K-Ras has 16 Asp and 10 Glu on the surface, and all are well exposed to the solvent (Figure S3). Four of them, including D12 of G12D, E62, D69, and D92, form direct contact to S-IIP upon ligand binding. Yet, **Diazo-G12Di-1** selectively ligated with the mutant D12. More strikingly, **Diazo-G12Di-1** did not modify K-Ras­(G12C), the most nucleophilic oncogenic mutant, in 2 h. By contrast, known K-Ras­(G12D) covalent inhibitors were either dual^13,14^ or multiple^16^ mutant-specific, such as **ML-G12Di-7** for G12C and G12D (Figure 1G). **Diazo-G12Di-1** emerged as the first molecule to exclusively react with G12D versus G12C.

K-Ras­(G12D)•**Diazo-G12Di-1** ligation was fast and selective at physiological pH buffer (20 mM HEPES pH 7.4, 150 mM NaCl, and 1 mM MgCl_2_). We found the covalent modification kinetics to be highly dependent on pH (Figure 1H). The structural change of K-Ras protein from physiological pH to lower pH is negligible (r.m.s.d. = 0.49 Å, K-Ras at pH 7.5 ^37^ and 6.5 ^38^). However, the chemoselective ligation was significantly faster at lower pH. The observation was in line with the fact that the protonation of diazo compounds is often rate-determining and supports the hypothetical proton transfer and ion pairing between the diazo group and Asp12. The d eprotonation-based ion-pairing mechanism poses an additional layer of selectivity for the target protein K-Ras­(G12D).

While an acidic environment accelerates the on-target engagement, it is also a potential liability for diazo compounds. We assessed its pH-dependent chemical stability in noncarboxylate aqueous buffer (HEPES) at 37 °C (Figure 1I). At a near physiological pH, **Diazo-G12Di-1** remained intact over 10 h. At pH 6.5, which mimics the extracellular environment of the tumor microenvironment, the compound also showed excellent stability, with a half-life greater than 24 h. In contrast, at pH 4.5, **Diazo-G12Di-1** degraded more rapidly, with a half-life of approximately 1 h. These results indicate that **Diazo-G12Di-1** is chemically stable under both physiological and pathologically relevant extracellular conditions but is susceptible to hydrolysis in highly acidic intracellular compartments.

We further demonstrated (Figure 2A) that **Diazo-G12Di-1**, as a covalent modifier, dramatically stabilized K-Ras­(G12D) protein with a melting temperature shift of +19 °C. Because of the structural similarity of the covalent adducts (Table S2), we assume that the critical interactions with K-Ras­(G12D) protein are conserved between the malolactone compound^13^ and **Diazo-G12Di-1**. Indeed, **Diazo-G12Di-1** blocked the interaction of K-Ras­(G12D) with the effector protein Raf Ras-binding domain (RafRBD) like other structurally relevant S-IIP inhibitors (Figure 2B), making it not only a covalent modifier but also a covalent inhibitor.

**Figure 2:**
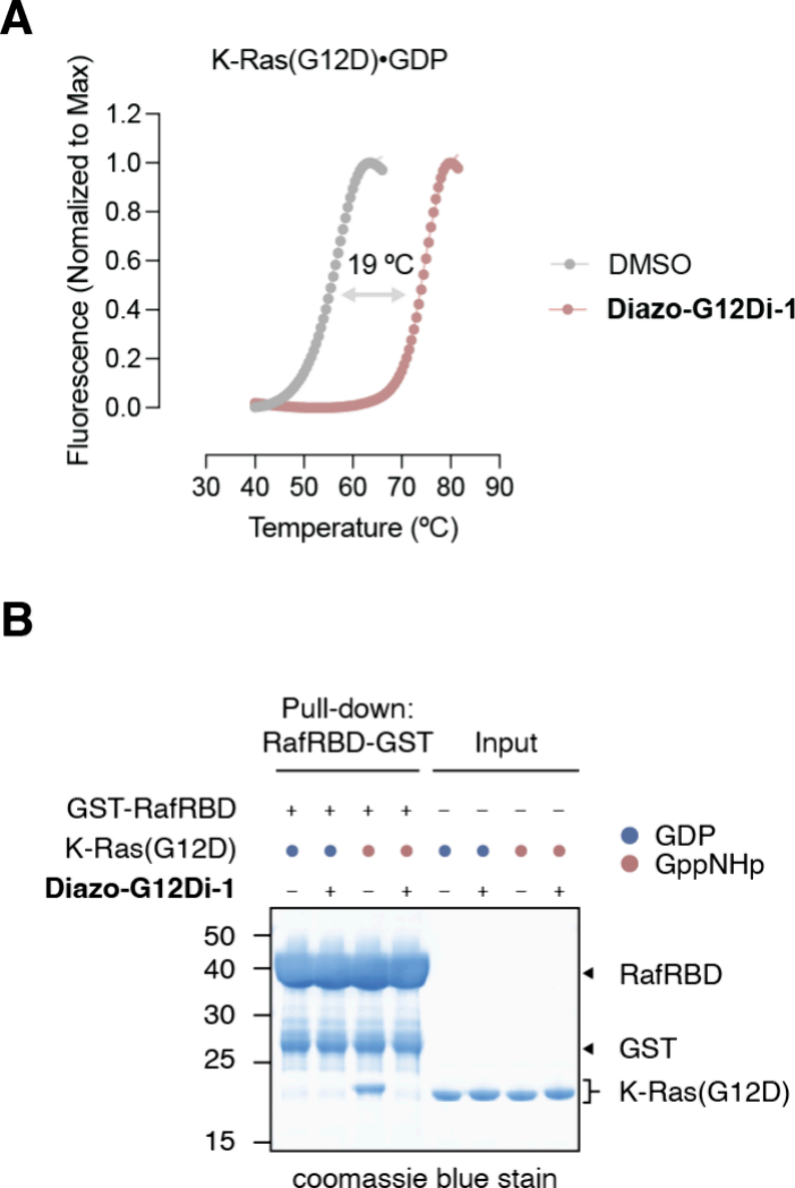
(A) **Diazo-G12Di-1**-induced stabilization of K-Ras­(G12D) by covalent modification. (B) Ras-RafRBD blockage by the **Diazo-G12Di-1**-induced conformation change.

The potent covalent inhibition of K-Ras­(G12D) by **Diazo-G12Di-1** is derived from both improved reversible binding affinity (MRTX1133, cf. weaker S-IIP binders^14,17^ ) and the correctly positioned reactive group. The unexpected access to the active-state K-Ras­(G12D) can be attributed to the unique MRTX1133 scaffold, especially the 3-hydroxy substitution on the naphthalene ring that forms a stabilizing hydrogen bond with Asp69 in the Switch-II Pocket.^13^

To understand the impact of the diazo warhead, we synthesized phenyl diazoacetamide **Diazo-G12Di-2** with the same MRTX1133 scaffold. **Diazo-G12Di-2** showed slightly faster GDP-state covalent modification kinetics but significantly slower GppNHp-state, which could be a result of the lower intrinsic reactivity and steric clash with the γ-phosphate of GTP or GTP-analogs (Figure 3). **Diazo-G12Di-3** and **Diazo-G12Di-4**, which derived from distinct S-IIP ligands GDC-6036 ^39^ (“off”-state K-Ras­(G12C) inhibitor) and BBO-8520 ^40^ (“on”-state K-Ras­(G12C) inhibitor), respectively, showed limited covalent modification of K-Ras­(G12D) protein despite the same α-diazoacetamide warhead (Figure 3). The structure–activity relationship underscores the importance of the right combination of the reversible binding S-IIP ligand and reactivity-matched covalent warhead. Taking the labeling efficiency of both nucleotide states into consideration, we chose **Diazo-G12Di-1** as the lead molecule in cellular assays.

**Figure 3:**
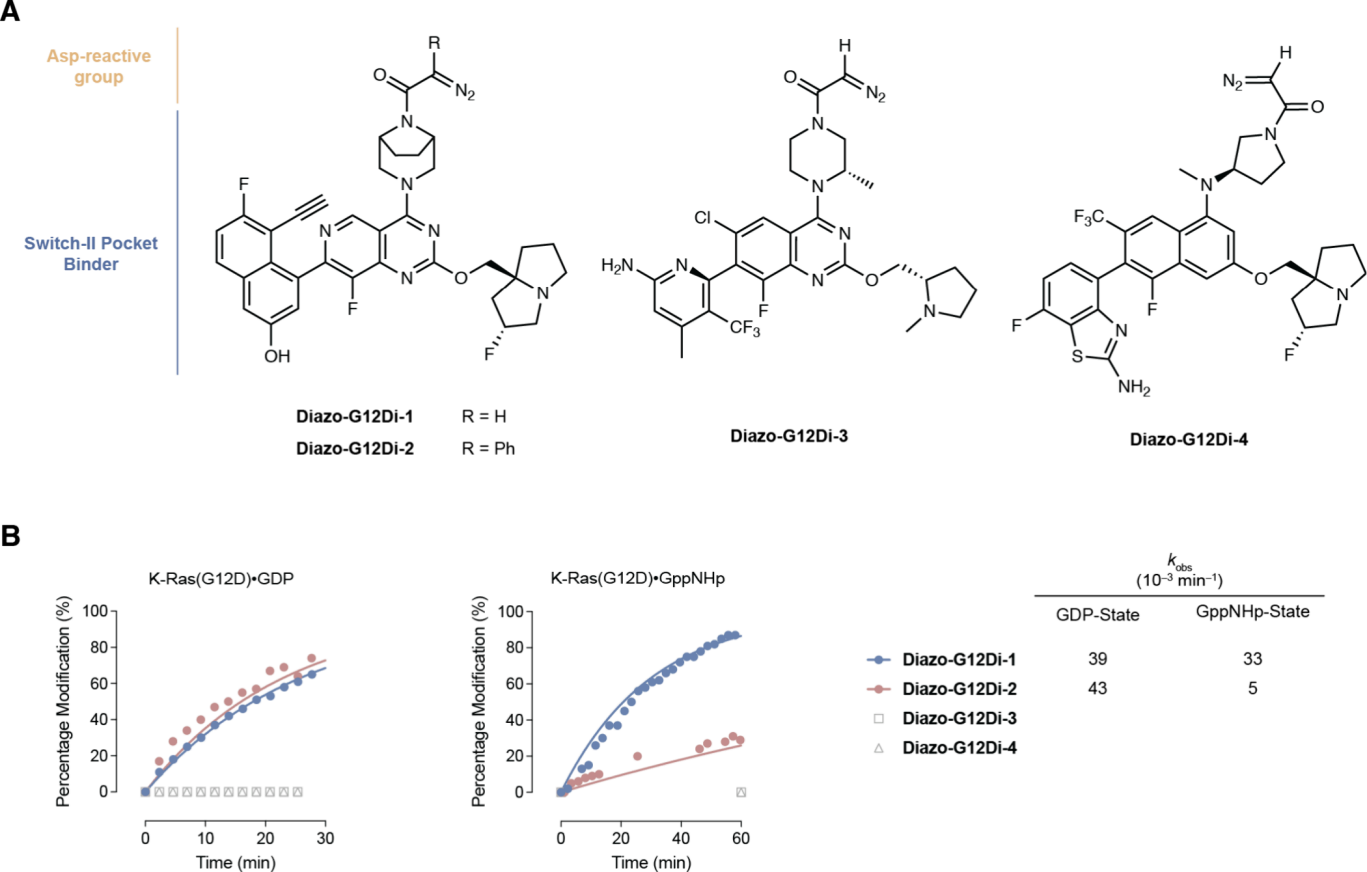
(A) Chemical structures of S-IIP ligand-derived α-diazoacetamide K-Ras­(G12D) modifiers. (B) Comparison of the covalent modification kinetics of α-diazoacetamides.

First, we asked the question of whether **Diazo-G12Di-1** covalently modifies endogenous K-Ras­(G12D) in cancer cells. We used a homozygous K-Ras­(G12D) mutant pancreatic adenocarcinoma cell line SW1990 treated with **Diazo-G12Di-1** for various durations up to 2 h (Figure 4A). At 1 μM concentration, **Diazo-G12Di-1** completely modified endogenous K-Ras­(G12D) protein and ablated ERK phosphorylation through Raf-MEK-ERK signaling.^41,42^ The covalent modification was judged by the disappearance of Asp12 epitope using a Ras­(G12D)-specific antibody and by the K-Ras protein band upshift in SDS-PAGE. In the time-course study, we found that the complete signaling suppression was achieved in 1 h. The faster *in vitro* covalent modification of K-Ras­(G12D) by **Diazo-G12Di-1**, compared to **ML-G12Di-7**, recapitulated its faster kinetics with the active-state recombinant K-Ras­(G12D) (Figure 4B). The GTP-bound “on”-state targeting in the cells was believed to contribute to the fast signaling inhibition observed.^10,43^ ERK inhibition was persistent 8 h after treatment.

**Figure 4:**
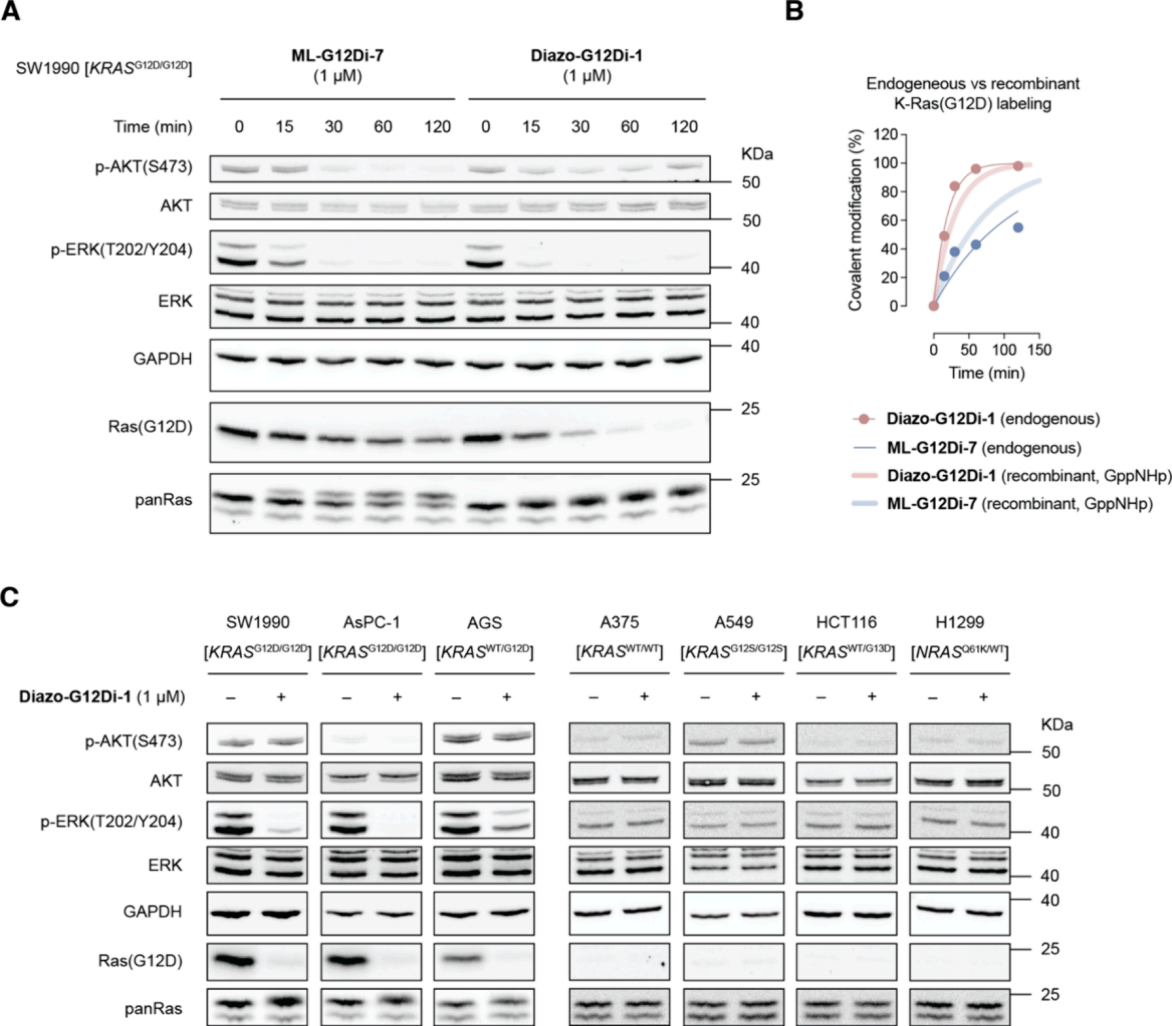
(A) Immunoblot-based time course of MAPK signaling suppression with K-Ras­(G12D) inhibitor **ML-G12Di-7** or **Diazo-G12Di-1** in the pancreatic cancer cell line SW1990. (B) Comparative covalent modification kinetics of endogenous K-Ras­(G12D) in SW1990 cells and purified recombinant K-Ras­(G12D)•GppNHp in an aqueous buffer. (C) Immunoblots of SW1990, AsPC-1, AGS, A375, A549, HCT116, and H1299 cells treated with DMSO or 1 μM **Diazo-G12Di-1** for 1 h.

In an immunoblot-based cell panel, we demonstrated the mutant-selective in-cell covalent inhibition of K-Ras­(G12D) by **Diazo-G12Di-1** (Figure 4C). **Diazo-G12Di-1** covalently labeled endogenous K-Ras protein in cancer cell lines harboring G12D mutation and inhibited the downstream signaling accordingly. By contrast, cell lines harboring a non-G12D K-Ras mutation or non-K-Ras mutation appeared to be insensitive to **Diazo-G12Di-1** treatment.

We next asked whether the signaling suppression induced by covalent inhibitor **Diazo-G12Di-1** resulted in cell growth inhibition. In human cancer cell lines harboring K-Ras­(G12D), such as SW1990, AsPC-1, and AGS, **Diazo-G12Di-1** showed 2–10-fold improved potency compared to malolactone **ML-G12Di-7**,^13^ appearing to be the most effective covalent S-IIP inhibitor for K-Ras­(G12D) reported to date. Meanwhile, **Diazo-G12Di-1** showed only moderate inhibition (GI_50_ > 10 μM) for non-K-Ras­(G12D) mutation cancer cell lines, such as H1299, HCT116, A549, and A375, demonstrating a desirable therapeutic window to spare K-Ras wild type in normal cells (Figure 5A).

**Figure 5:**
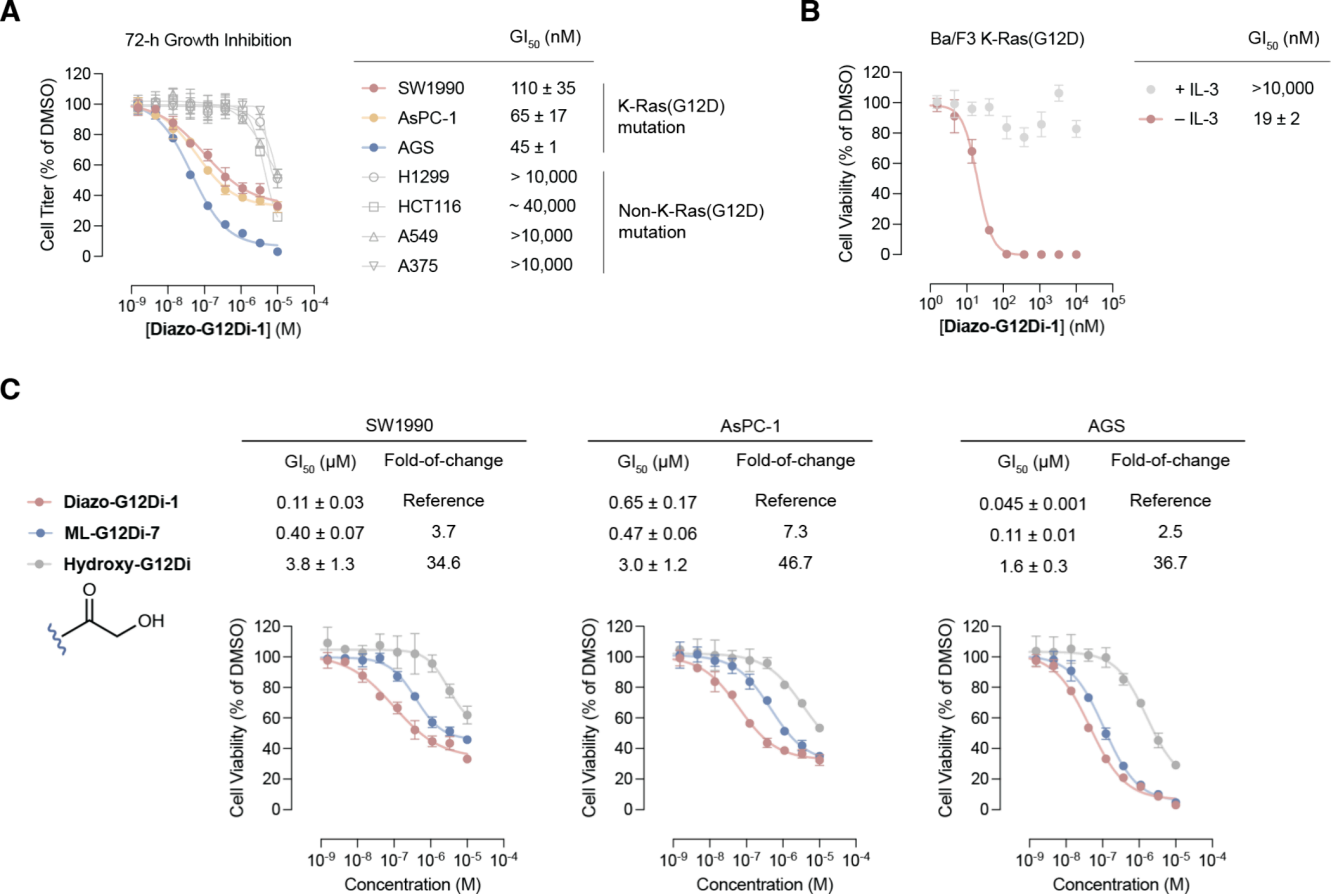
(A) Human cancer cell growth inhibition by **Diazo-G12Di-1**. (B) Ba/F3 K-Ras­(G12D) cell growth inhibition by **Diazo-G12Di-1**. (C) Comparison of cell growth inhibition induced by covalent or noncovalent K-Ras­(G12D) inhibitors.

To confirm if the cell growth inhibition we observed was due to the on-target inhibition of oncogenic K-Ras signaling, we used the K-Ras­(G12D)-transformed mouse pro-B cell line Ba/F3.^44^ The parental Ba/F3 cell growth is dependent on interleukin-3 (IL-3), while the transformed cells become independent of IL-3 but dependent on the transduced driver oncogene K-Ras­(G12D). We treated Ba/F3 K-Ras­(G12D) cells with **Diazo-G12Di-1** and observed potent cell growth inhibition (Figure 5B, GI_50_ 19 ± 2 nM). **Diazo-G12Di-1** is 4 times more potent compared to malolactone-based **ML-G12Di-7**.^13^ When cotreated with IL-3, the cell growth became independent of **Diazo-G12Di-1**, which suggested that the observed Ba/F3 K-Ras­(G12D) cell growth inhibition was a result of on-target inhibition of the transduced oncogene.

Finally, we sought to dissect the contribution of covalent engagement to the overall potency of **Diazo-G12Di-1**. We synthesized a control **Hydroxy-G12Di**, which is a common degradation product of **Diazo-G12Di-1**, through acid-mediated hydrolysis and a noncovalent S-IIP inhibitor. Although **Hydroxy-G12Di** showed inhibition of the growth of K-Ras­(G12D) mutation cell lines, the noncovalent inhibitor was 30–50-fold less potent than its covalent precursor **Diazo-G12Di-1** (Figure 5C). These results, along with our earlier report^13^ on malolactone-based covalent G12D inhibitors, suggested that strong noncovalent binding and efficient and selective Asp12 reactivity are critical to achieve potent inhibition in cancer cells.

To conclude, our data have revealed the first aspartic-acid-targeting covalent inhibitor based on α-diazoacetamides. The diazo-carboxy ligation has been known in chemistry for more than a century and mostly used in promiscuous acid labeling reactions. Here we show that the reactivity, coupled with a reversible binding ligand, could be harnessed to selectively modify the acquired aspartic acid in the K-Ras­(G12D) mutation in live cells. Our discovery has added one potent candidate to the growing inhibitor repertoire against the most common oncogenic mutation in human cancer with no approved therapies. The chemistry reported here holds the potential to inspire future therapeutic agent development against aspartic or glutamic acids in the human cancer proteome.

## Supplementary Material



## Notes

Supplementary figures (Figures S1–S3); supplementary tables (Tables S1–S4); methods of biochemical or cellular experiments; synthetic procedures; and characterization details of compounds, including ^1^H, ^19^F NMR and mass spectrometry (PDF)

## #

Department of Biological Chemistry and Molecular Pharmacology, Blavatnik Institute, Harvard Medical School, Boston, Massachusetts 02115, United States
