# Supplementary material for: Denitrogenative Alkylation of K‑Ras(G12D) Inhibits Oncogenic Signaling in Cancer Cells
Source: J Am Chem Soc. 2025 Jun 30;147(28):24785–92. doi: 10.1021/jacs.5c06745 (PMC12272675; doi:10.1021/jacs.5c06745)

Supporting Information

Denitrogenative alkylation of K-Ras(G12D) inhibits oncogenic signaling in cancer cells

Qinheng Zheng[1], and Kevan M. Shokat[1,2]*

[1]Department of Cellular and Molecular Pharmacology and Howard Hughes Medical Institute, University of California, San Francisco, California 94158, United States. [2]Department of Chemistry, University of California, Berkeley, California 94720, United States.

*Correspondence: kevan.shokat@ucsf.edu

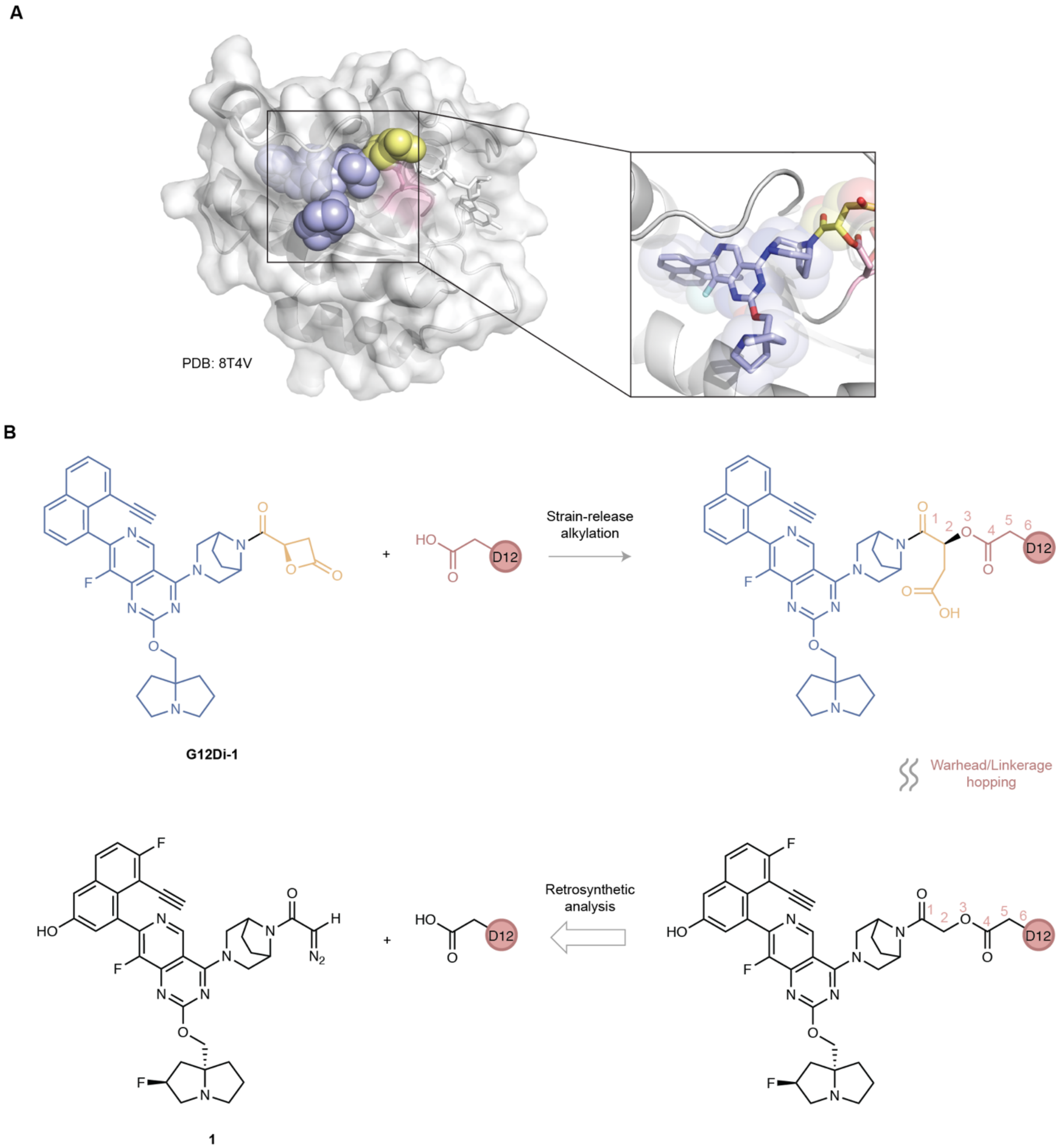

Figure S1. (A) Protein-ligand interaction of malolactone-based K-Ras(G12D) covalent inhibitor G12Di-1. (B) Design of α-diazoacetamide-based K-Ras(G12D) covalent inhibitor via warhead hopping and retrosynthetic analysis.

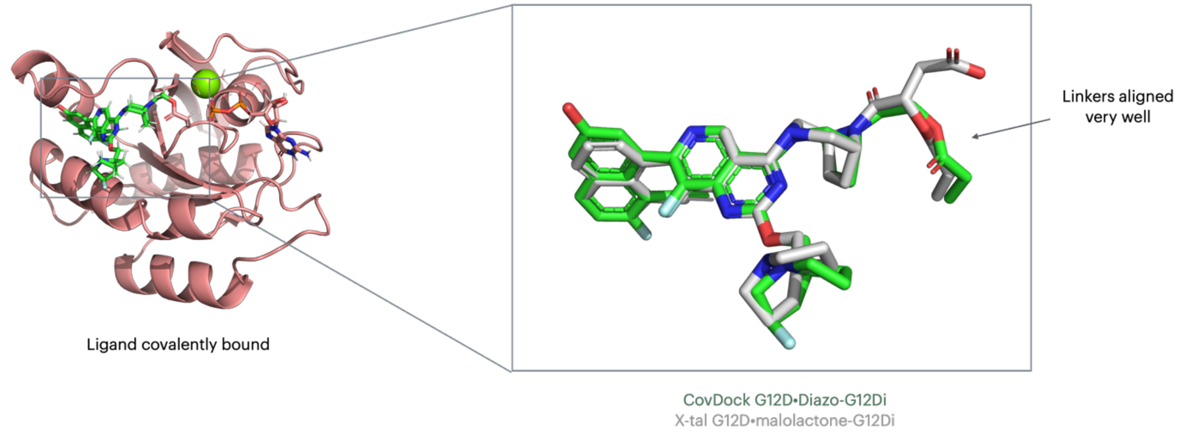

Figure S2. Alignment of Diazo-G12Di (docked) and Malolactone-G12Di (PDB Code 8T4V) Structures.

A

```
>sp|P01116-2|RASK_HUMAN Isoform 2B of GTPase KRas OS
        10         20         30         40         50         60
MTEYK LVVVG AGGVG KSALT IQLIQ NHFVD EYDPT IEDSY RKQVV IDGET CLLDI LDTAG
        70         80         90        100        110        120
QEEYS AMRDQ YMRTG EGFLC VFAIN NTKSF EDIHH YREQI KRVKD SEDVP MVLVG NKCDL
       130        140        150        160        170        180
PSRTV DTKQA QDLAR SYGIP FIETS AKTRQ GVDDA FYTLV REIRK HKEKM SKDGK KKKKK

SKTKC VIM
```

B **C**

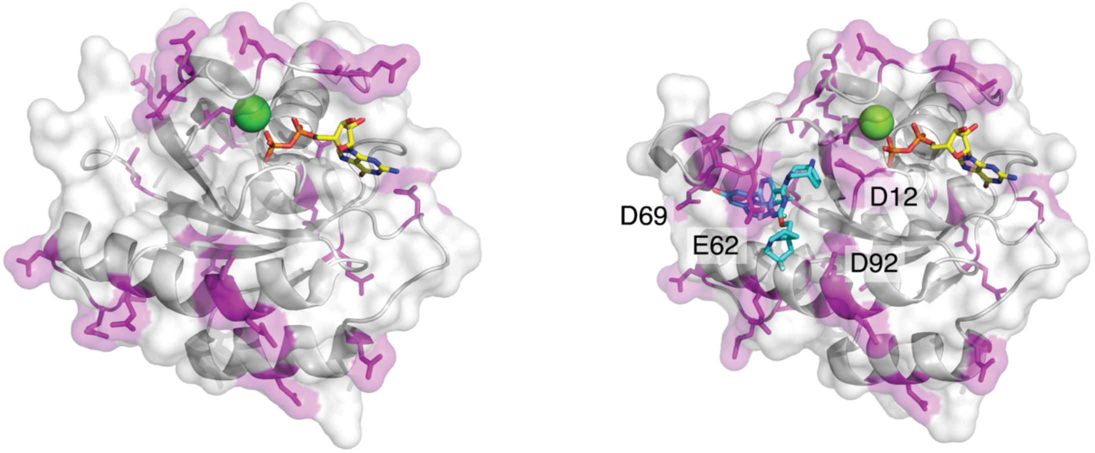

K-Ras
PDB 4PLK

K-Ras(G12D)•MRTX1133
PDB 7RPZ

Figure S3. (A) Sequence of K-Ras4B. (B) Structure of K-Ras•GDP with acidic amino acid residues highlighted in magenta. (C) Structure of K-Ras(G12D)•GDP•MRTX1133 with acidic amino acid residues highlighted in magenta.

Table S1. Alignment of K-Ras•GDP structures in the PDB database.

K-Ras	PDB Entry	r.m.s.d. (Å)	TM-score	Aligned Residues
Wildtype	4LPK	-	-	-
Wildtype	6MBT	0.77	0.98	156
Wildtype	6OBE	0.81	0.98	155
G12D	5US4	1.01	0.98	155
G12C	4L8G	0.39	0.99	155
G12C	4LRW	0.56	0.97	154
G12C	4LDJ	0.63	0.98	156
G12S	7TLK	0.31	1	156
G12R	4QL3	0.62	0.98	156
G12V	7C40	0.52	0.99	156
G12V	4TQ9	0.74	0.98	156
G13D	4TQA	0.77	0.98	155
Q61H	6MNX	0.87	0.97	156
Q61L	4WA7	0.37	0.99	155

Table S2. Alignment of K-Ras(G12D)•inhibitor co-crystal structures in the PDB database.

Ligand	PDB #	Complex (Binding Mode)	RMSD (Å) to 7RPZ
**Diazo-G12Di-1 (This report)**	N/A	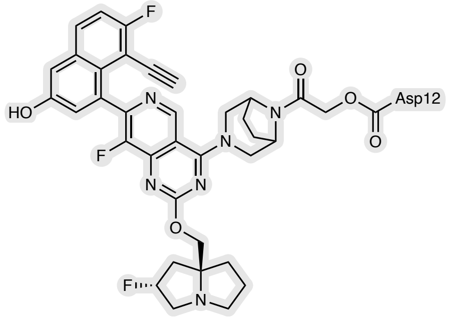 (Covalent)	N/A
**MRTX1133**	7RPZ	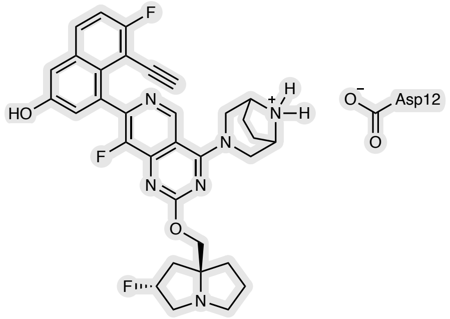 (Non-covalent)	Reference
**G12Di-1 (ML-G12Di-1)**	8T4V	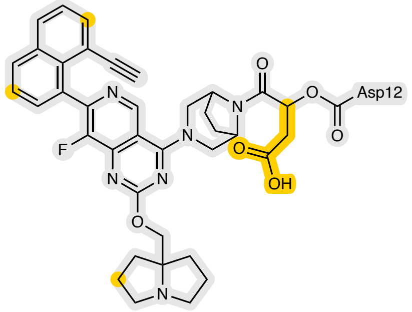 (Covalent)	0.375
**YK-8S**	8JHL	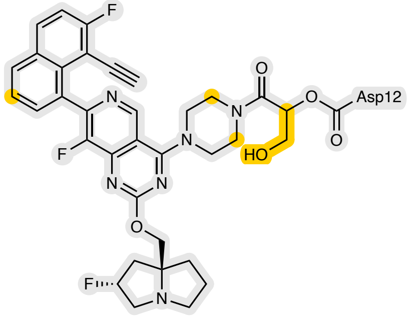 (Covalent)	0.357

Table S3. List of antibodies.

Target	Species	Supplier	Identifier	Dilution
p-AKT (S473)	Rabbit	Cell Signaling Technology	4060	1:1,000
AKT	Rabbit	Cell Signaling Technology	2920	1:1,000
p-ERK (T202/Y204)	Rabbit	Cell Signaling Technology	9101	1:1,000
ERK	Rabbit	Cell Signaling Technology	4695	1:1,000
Ras(G12D)	Rabbit	Invitrogen (Thermo)	MA5-36256	1:1,000
Pan-Ras	Rabbit	Abcam	108602	1:5,000
GAPDH	Mouse	Proteintech	60004-1-Ig	1:50,000

Table S4. List of buffer compositions.

Name	Composition
RIPA Buffer	25 mM Tris 7.4, 150 mM NaCl, 0.1% SDS, 1% NP-40, 0.5% Sodium Deoxycholate
5X SDS Loading Buffer	250 mM Tris 6.8, 500 mM DTT, 10% w/v SDS, 0.1% w/v Bromophenol Blue, 50% Glycerol
1X TOWBIN Transfer Buffer	250 mM Tris, 192 mM Glycine, pH 8.3
Lysis Buffer	20 mM Tris 8.0, 500 mM NaCl, 5 mM Imidazole
Elution Buffer	20 mM Tris 8.0, 300 mM NaCl, 300 mM Imidazole
TEV Cleavage Buffer	20 mM Tris 8.0, 300 mM NaCl, 1 mM EDTA
SEC Buffer	20 mM HEPES 7.5, 150 mM NaCl, 1 mM $MgCl_2$
Nucleotide Exchange Buffer	20 mM HEPES 7.5, 150 mM NaCl, 1 mM $MgCl_2$, 1 mM DTT

Covalent docking

The structure of K-Ras(G12D)•GDP bound to Switch-II Pocket ligand MRTX1133 (PDB ID: 7RPZ) was prepared using the standard Schrödinger Protein Preparation protocol. Water molecule O357 was manually removed to make room for the docking of covalent warheads. A 20-Å cubic receptor grid was generated centered at the native ligand MRTX1133. Ligand 3D coordinates were generated from SMILES strings using LigPrep.

The covalent compounds were first docked into the Switch-II Pocket using GlideDock to validate the reversible binding in the presence of covalent warheads. A standard covalent docking protocol (CovDock) was applied using a customized reaction type. Docking scores and structures were summarized and reported in the Supporting Information.

Recombinant protein expression and purification

K-Ras wildtype and mutant cyslight DNA sequences encoding human K-Ras cyslight (C51S/C80L/C118S, a.a. 1–169), human K-Ras(G12D) cyslight (G12D/C51S/C80L/C118S, a.a. 1–169), human K-Ras(G12C) cyslight (G12C/C51S/C80L/C118S, a.a. 1–169), human K-Ras(G12E) cyslight (G12E/C51S/C80L/C118S, a.a. 1–169), human K-Ras(G12S) cyslight (G12S/C51S/C80L/C118S, a.a. 1–169), human K-Ras(G12V) cyslight (G12V/C51S/C80L/C118S, a.a. 1–169), human K-Ras(G12R) cyslight (G12R/C51S/C80L/C118S, a.a. 1–169), human K-Ras(G13C) cyslight (G13C/C51S/C80L/C118S, a.a. 1–169), human K-Ras(G13D) cyslight (G13D/C51S/C80L/C118S, a.a. 1–169), human K-Ras(Q61H) cyslight (C51S/Q61H/C80L/C118S, a.a. 1–169), human K-Ras(Q61K) cyslight (C51S/Q61K/C80L/C118S, a.a. 1–169), and human K-Ras(Q61R) cyslight (C51S/Q61R/C80L/C118S, a.a. 1–169) were codon optimized, synthesized by Twist Biosciences and cloned into pJExpress411 vector between NdeI and XhoI sites. The resulting construct contains N-terminal 6xHis tag and a Tobacco Etch Virus (TEV) protease cleavage site (ENLYFQ^G). The proteins were expressed and purified following previously reported protocols[1]. Briefly, chemically competent BL21(DE3) cells were transformed with the corresponding plasmid and grown on LB agar plates containing 50 μg ml^{-1} kanamycin. A single colony was used to inoculate a culture at 37 °C, 220 rpm in terrific broth containing 50 μg mL^{-1} kanamycin. When the optical density (OD_{600}) reached 0.6, the culture temperature was reduced to 18 °C, and protein expression was induced by the addition of isopropyl β-d-1-thiogalactopyranoside to 1 mM. After 16 h at 18 °C, the cells were pelleted by centrifugation (6,500*g*, 10 min) and lysed in lysis buffer (20 mM Tris 8.0, 500 mM NaCl and 5 mM imidazole) with a high-pressure homogenizer (Microfluidics). The lysate was clarified by high-speed centrifugation (19,000*g*, 15 min) and the supernatant was used in subsequent purification by immobilized metal affinity chromatography. His-TEV-tagged protein was captured with Co-TALON resin (Clonetech, Takara Bio, 2 ml slurry per liter culture) at 4 °C for 1 h with constant end-to-end mixing. The loaded beads were then washed with lysis buffer (50 ml per liter culture), and the protein was eluted with elution buffer (20 mM Tris 8.0, 300 mM NaCl and 300 mM imidazole). To this protein solution was added His-tagged TEV protease (Berkeley QB3 MacroLab, 0.05 mg TEV per milligram Ras protein) and GDP (1 mg per milligram Ras protein), and the mixture was dialyzed against TEV cleavage buffer (20 mM Tris 8.0, 300 mM NaCl, 1 mM

ethylenediaminetetraacetic acid and 1 mM DTT) at 4 °C using a 10 K molecular weight cutoff (MWCO) dialysis cassette until liquid chromatography (LC)–MS analysis showed full cleavage (typically 16–24 h). $MgCl_2$ was added to a final concentration of 5 mM, and the mixture was incubated with 1 ml Ni-NTA (Qiagen) beads at 4 °C for 1 h to remove TEV protease, any residual His-tagged proteins and peptides. The protein solution was diluted 1:10 v/v with 20 mM Tris 8.0 and further purified with anion exchange chromatography (HiTrapQ column, GE Healthcare Life Sciences) using a NaCl gradient of 50 mM to 500 mM in 20 mM Tris 8.0. Nucleotide loading was performed by mixing the ion exchange-purified protein with an excess of GDP (5 mg per liter culture) or GppNHp (5 mg per liter culture) and 5 mM ethylenediaminetetraacetic acid at 23 °C for 30 min. The reaction was stopped by the addition of $MgCl_2$ to 10 mM. For GppNHp, an additional calf intestinal phosphatase (CIP) treatment was performed as follows to ensure high homogeneity of the loaded nucleotide. The protein buffer was exchanged into phosphatase buffer (32 mM Tris 8.0, 200 mM ammonium sulfate and 0.1 mM $ZnCl_2$) with a HiTrap Desalting Column (GE Healthcare Life Sciences). To the buffer-exchanged protein solutions, GppNHp was added to 5 mg mL^{-1}, and calf intestinal phosphatase (NEB) was added to 10 U mL^{-1}. The reaction mixture was incubated on ice for 1 h, and $MgCl_2$ was added to a final concentration of 20 mM. After nucleotide loading, the protein was concentrated using a 10 K MWCO centrifugal concentrator (Amicon-15, Millipore) to 20 mg ml^{-1} and purified by size exclusion chromatography on a Superdex 75 10/300 GL column (GE Healthcare Life Sciences). Fractions containing pure Ras protein were pooled and concentrated to 20 mg mL^{-1} and stored at −80 °C. In our hands, this protocol gives a typical yield of 30 mg per liter of culture.

Cell culture

AsPc-1 (CRL-1682), SW1990 (CRL-2172), H1299 (or NCI-H1299, CRL-5803), HCT-116 (CCL-247), A549 (CRM-CCL-185), A375 (or A-375, CRL-1619), HEK293 (CRL-1573) cells were obtained from ATCC and maintained in high-glucose (4.5 g l^{-1}) DMEM (Gibco 11995073) supplemented with 4 mM L-glutamine, 1 mM sodium pyruvate and 10% heat-inactivated fetal bovine serum (FBS; Axenia Biologix). AGS (CRL-1739) cells were obtained from ATCC and maintained in Ham's F12-K (Gibco 21127022) supplemented with 10% heat-inactivated FBS (Axenia Biologix).

Ba/F3 cells were obtained from ATCC and were maintained in RPMI-1640 (Gibco 11875093) supplemented with 10% heat-inactivated FBS (Axenia Biologix) and 10 ng mL^{-1} recombinant mouse IL-3 (Gibco PMC0031).

Cells were passed for at least two generations after cryorecovery before they were used for assays. All cell lines were tested mycoplasma negative using MycoAlert Mycoplasma Detection Kit (Lonza LT07-318).

Gel electrophoresis and immunoblotting

Cells were treated with compounds at 40–60% confluency at a final DMSO concentration no higher than 1%. At the end of the treatment period, cells were chilled on ice. Unless otherwise indicated, adherent cells were washed once with ice-cold PBS (1 ml), scraped with a spatula, and pelleted by centrifugation (500*g*, 5 min). Suspension cells were pelleted by centrifugation (500*g*, 5 min), washed with 1 ml ice-cold PBS, and pelleted again. Cells were lysed in

radioimmunoprecipitation assay buffer (RIPA) buffer supplemented with protease and phosphatase inhibitors (mini cOmplete and phosSTOP, Roche) on ice for 10 min. Concentrations of lysates were determined with protein bicinchoninic acid (BCA) assay (Thermo Fisher) and adjusted to 2 mg mL^{-1} or lowest available concentration with additional RIPA buffer. Samples were mixed with 5× sodium dodecyl sulfate (SDS) loading dye and denatured at 95 °C for 5 min

Unless otherwise noted, SDS–polyacrylamide gel electrophoresis was run with Novex 12% Bis-Tris gel (Invitrogen) in MOPS running buffer (Invitrogen) at 200 V for 60 min following the manufacturer's instructions. Proteins were transferred onto 0.2-μm nitrocellulose membranes using a semi-dry transfer device (Invitrogen iBlot 3). Membranes were blocked in 5% bovine serum albumin (BSA)–tris-buffered saline-Tween 20 (TBST) for 1 h at 23 °C. Primary antibody binding was performed with the indicated antibodies diluted in 5% BSA–TBST at 4 °C for at least 16 h. After washing the membrane three times with TBST (5 min each wash), secondary antibodies (goat anti-rabbit IgG-IRDye 800 and goat anti-mouse IgG-IRDye 680, Li-COR) were added as solutions in 5% BSA–TBST at the dilutions recommended by the manufacturer. Secondary antibody binding was allowed to proceed for 1 h at 23 °C. The membrane was washed three times with TBST (5 min each wash) and imaged on a ChemiDoc MP imaging system (Bio-Rad).

Preparation of MSCV

MSCV-Puro plasmid was obtained from Addgene (Plasmid #68469). Full length human *KRAS* genes (G12D, c.35G>A) were cloned into MSCU-Puro plasmid between the BglII and XhoI sites. Transfection-grade plasmids were prepared using ZymoPure II Plasmid Midiprep kit. EcoPack 293 cells (Takara Bio) were plated in six-well plates (3×10^5 mL^{-1}, 2 mL). The next day, cells were transfected with 2.5 μg MSCV plasmid using Lipofectamine 3000 following the manufacturer's instructions. The cells were incubated for 66 h, and then the virus-containing supernatants were collected and passed through a 0.22-μm syringe filter. The collected virus was used immediately for spinfection of Ba/F3 cells or stored at −80 °C.

Generation of stable Ba/F3 transductants

One milliliter of MSCV-containing supernatant (vide supra) was added to one well of a six-well plate containing 1×10^6 Ba/F3 cells in 1 ml of medium composed of 60% RMPI 1640, 40% heat-inactivated fetal bovine serum (FBS), 10 ng mouse IL-3 and 4 μg polybrene. Cells were spinfected by centrifugation at 2,000*g* for 90 min at room temperature and then placed in the incubator for 24 h. After 1 day, the cells were diluted into 10 mL culture medium (RPMI 1640 + 10% heat-inactivated FBS, 10 ng mL^{-1} mouse IL-3) and recovered for a second day after spinfection. On the third day after spinfection, cells were pelleted at 500*g* for 5 min and resuspended in 10 mL selection medium (RPMI 1640 + 10% heat-inactivated FBS, 10 ng mL^{-1} mouse IL-3 and 1.25 μg ml^{-1} puromycin). Cells were maintained under puromycin selection for 4–7 days, splitting as required to maintain density <2×10^6 cells mL^{-1}. After 7 days, cells were pelleted, washed once with IL-3 free culture medium (RPMI 1640 + 10% heat-inactivated FBS) and pelleted again before resuspending at 2–4 × 10^5 cells mL^{-1} in IL-3 free culture medium. Cells were maintained under these conditions for 7 days, passaging as needed

to maintain density <2 × 10^6 cells mL^{-1}. Growth was monitored (Countess II Cell Counter) over these 7 days to confirm that an IL-3 independent population has been achieved.

Differential scanning fluorimetry

The protein of interest was diluted with SEC buffer (20 mM HEPES 7.5, 150 mM NaCl and 1 mM $MgCl_2$) to 2 µM. This solution was dispensed into wells of a white 96-well polymerase chain reaction (PCR) plate in triplicate (25 µL per well). Fluorescence was measured at 0.5 °C temperature intervals every 30 s from 25 °C to 95 °C on a Bio-Rad CFX96 qPCR system using the FRET setting. Each dataset was normalized to the highest fluorescence and the normalized fluorescence reading was plotted against temperature in GraphPad Prism 10. T_m values were determined as the temperature(s) corresponding to the maximum of the first derivative of the curve. Proteins crosslinked with small molecules were desalted using Zeba Spin Desalting Columns (Thermo) before differential scanning fluorimetry T_m measurement.

Detection of covalent modification of K-Ras by whole-protein MS

Test compounds were prepared as 100× stock solutions in DMSO. K-Ras proteins were diluted with SEC buffer (20 mM HEPES 7.5, 150 mM NaCl and 1 mM $MgCl_2$) to 1 µM. In a typical reaction, 1 µL 100× compound stock was mixed with 99 µL diluted K-Ras protein, and the resulting mixture was incubated for the desired amount of time. The extent of modification was assessed by electrospray MS using a Waters Xevo G2-XS system equipped with an Acquity UPLC BEH C4 1.7 µm column. The mobile phase was a linear gradient of 5–95% acetonitrile/water + 0.05% formic acid. For kinetic measurements, a 2× compound solution was first prepared in SEC buffer, which was then mixed with 400 nM K-Ras-G12D protein at 1:1 (v/v) ratio. Elapsed time was calibrated to the first injection.

Cell viability assay

Cells were seeded into 96-well white flat-bottom plates (1,000 cells per well) (Greiner Bio-One, 655083) and incubated overnight. Cells were treated with the indicated compounds in a nine-point threefold dilution series (100 µL final volume) and incubated for 72 h. Cell viability was assessed using a commercial CellTiter-Glo (CTG) luminescence-based assay (Promega). The 96-well plates were equilibrated to room temperature before the addition of diluted CTG reagent (100 µL) (1:4 CTG reagent:PBS, containing 1% Triton X-100). Plates were placed on an orbital shaker for 30 min before recording luminescence using a Spark 20M (Tecan) plate reader.

Chemical Synthesis

General Experiment Procedure

All reactions were performed in oven-dried glassware fitted with rubber septa under a positive pressure of argon, unless otherwise noted. Air- and moisture-sensitive liquids were transferred via syringe. Solutions were concentrated by rotary evaporation at or below 40 °C. Analytical thin-layer chromatography (TLC) was performed using glass plates pre-coated with silica gel (0.25-mm, 60-Å pore size, 230−400 mesh, Merck KGA) impregnated with a fluorescent indicator (254 nm). TLC plates were visualized by exposure to ultraviolet light (UV), then were stained by submersion in a 10% solution of phosphomolybdic acid (PMA) in ethanol or an 2% aqueous

solution of potassium permanganate followed by brief heating on a hot plate. Flash column chromatography was performed with Teledyne ISCO CombiFlash EZ Prep chromatography system, employing pre-packed silica gel cartridges (Teledyne ISCO RediSep).

Solvents and Reagents

Anhydrous solvents were dispensed from a solvent purification system under argon. Unless specified below, all chemical reagents were purchased from Sigma-Aldrich, AK Scientific, Combi-Blocks, Chemscene (**Diazo-G12Di-4** piperazine precursor), and Advanced ChemBlock (MRTX1133, **Diazo-G12Di-3** piperazine precursor). Stable isotope regents were purchased from Cambridge Isotope Laboratories.

Instrumentation

Proton nuclear magnetic resonance (^{1}H NMR) spectra, carbon nuclear magnetic resonance (^{13}C NMR) spectra, and fluorine nuclear magnetic resonance (^{19}F NMR) spectra were recorded on Bruker AvanceIII HD instrument (400 MHz/100 MHz/376 MHz) or Varian Inova 600 instrument (600 MHz/126 MHz/564 MHz) at 23 °C. Proton chemical shifts are expressed in parts per million (ppm, δ scale) and are referenced to residual protium in the NMR solvent ($CHCl_3$: δ 7.26, D_2HCOD: δ 3.31, CD_2HCOCD_3: δ 2.05, CD_2HSOCD_3: δ 2.50). Carbon chemical shifts are expressed in parts per million (ppm, δ scale) and are referenced to the carbon resonance of the NMR solvent ($CDCl_3$: δ 77.0, CD_3OD: δ 49.0, $CD_3\underline{C}OCD_3$: δ 206.26, CD_3SOCD_3: δ 39.52). Fluorine chemical shifts are expressed in parts per million (ppm, δ scale) and are referenced to an internal standard of trifluoroacetic acid (–76.55 ppm). Data are represented as follows: chemical shift, multiplicity (s = singlet, d = doublet, t = triplet, q = quartet, dd = doublet of doublets, dt = doublet of triplets, m = multiplet, br = broad, app = apparent), integration, and coupling constant (*J*) in Hertz (Hz). Accurate (high-resolution) mass spectra were obtained using a Waters Xevo G2-XS time-of-flight mass spectrometer.

Monitoring Reaction Progress by LC-MS

When LC-MS analysis of the reaction mixture is indicated in the procedure, it was performed as follows. An aliquot (1 µL) of the reaction mixture (or the organic phase of a mini-workup mixture) was diluted with 100 µL 1:1 acetonitrile:water. 1 µL of the diluted solution was injected onto a Waters Acquity UPLC BEH C18 1.7 µm column and eluted with a linear gradient of 5–95% acetonitrile/water (+0.1% formic acid) over 3.0 min. Chromatograms were recorded with a UV detector set at 254 nm and a time-of-flight mass spectrometer (Waters Xevo G2-XS).

Synthesis of α-diazoacetamides

α-Diazoacetamides were synthesized using a modular approach[2-4] by combining a nucleophilic Switch-II Pocket binding ligand with an NHS-diazoacetate in a mixture of DMSO and water. Water was found critical to mediate the amide bond formation. Flash column chromatography was ideal to purify the diazoacetamide product while typical preparative HPLC mobile phase (supplemented with either TFA or formic acid) led to acid-mediated hydrolysis of the diazo compounds. Stable isotope labeled Compounds [^{2}H]**1**, [$^{15}N_2$]**1**, and [$^{14}C_2$]**1** were synthesized

using isotope labeled NHS-diazoacetate[5]. Isotope labeled compounds were characterized by LC-MS.

Diazo-G12Di-1

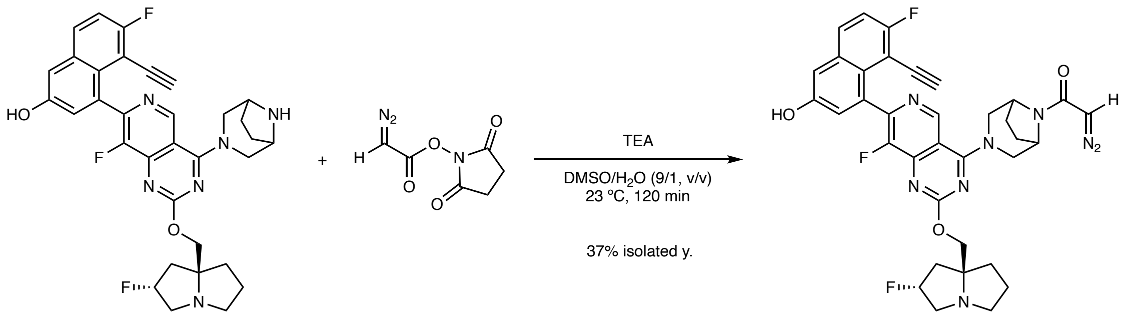

Diazo-G12Di-1

A 4-mL vial equipped with a stir bar was charged with MRTX1133 [Advanced Chemblocks, 4-(4-((1*R*,5*S*)-3,8-diazabicyclo[3.2.1]octan-3-yl)-8-fluoro-2-(((2*R*,7a*S*)-2-fluorotetrahydro-1*H*-pyrrolizin-7a(5*H*)-yl)methoxy)pyrido[4,3-*d*]pyrimidin-7-yl)-5-ethynyl-6-fluoronaphthalen-2-ol, 20.0 mg, 0.033 mmol], and 2,5-dioxopyrrolidin-1-yl 2-diazoacetate[3] (6.8 mg, 0.040 mmol, 1.2 equiv). DMSO (0.45 mL) and deionized water (0.05 mL) were added sequentially to result a dark brown homogeneous solution. Triethylamine (6.7 mg, 0.067 mmol, 2 equiv) was added via pipette. The reaction was stirred at room temperature for 2 h, and the mixture was directly loaded onto a silica cartridge before purification by flash column chromatography (0–30% MeOH-DCM, 12-g Gold RediSep(R) Rf column, Teledyne ISCO, Lincoln, NE) to give a brown solid (8.2 mg, 0.012 mmol, 37% yield). Accurate MS calculated for $C_{35}H_{32}F_3N_8O_3$ $[M + H]^+$ 669.2549, found 669.2659. ^{1}H NMR (400 MHz, DMSO) δ 10.22 (s, 1H), 9.05 (s, 1H), 7.98 (dd, *J* = 9.2, 5.9 Hz, 1H), 7.46 (t, *J* = 9.0 Hz, 1H), 7.40 (d, *J* = 2.6 Hz, 1H), 7.19 (d, *J* = 2.5 Hz, 1H), 6.10 (s, 1H), 5.42 – 5.16 (m, 1H), 4.66 – 4.26 (m, 4H), 4.15 (d, *J* = 10.4 Hz, 1H), 4.05 (d, *J* = 10.5 Hz, 1H), 3.95 (d, *J* = 1.1 Hz, 1H), 3.76 – 3.55 (m, 2H), 3.22 – 2.98 (m, 3H), 2.92 – 2.78 (m, 1H), 2.59 (s, 1H), 2.18 – 1.98 (m, 3H), 1.81 (t, *J* = 11.0 Hz, 7H). ^{19}F NMR (376 MHz, DMSO) δ -110.60 (dd, *J* = 11.9, 6.6 Hz), -139.64 – -139.86 (m), -172.87 (m).

Diazo-G12Di-2

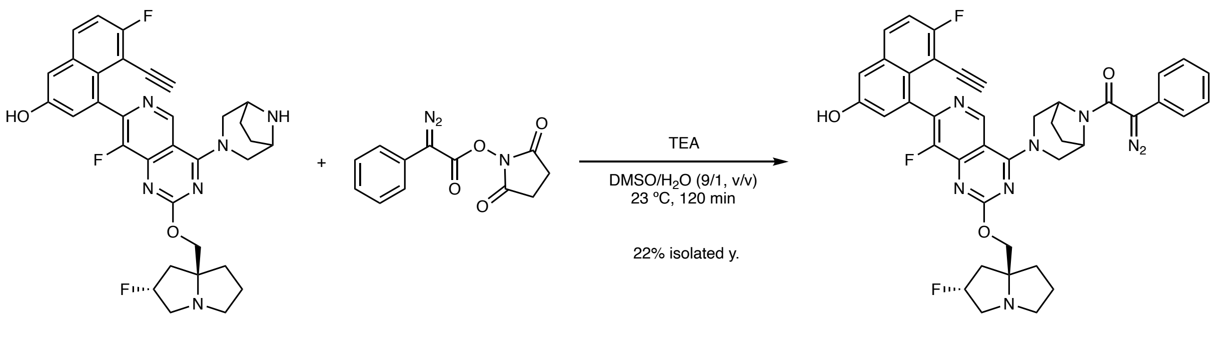

Diazo-G12Di-2

A 1-dram vial equipped with a stir bar was charged with MRTX1133 (30.0 mg, 0.0499 mmol) and 2-(2,5-dioxopyrrolidin-1-yl)oxy-2-oxo-1-phenyl-ethanediazonium[3] (25.9 mg, 0.0999 mmol). DMSO (0.45 mL) and water (0.05 mL) were added sequentially to result a dark brown homogeneous solution. Triethylamine (10.1 mg, 0.0999 mmol) was added via pipette. The reaction was stirred at room temperature overnight. The reaction mixture was directly loaded onto a silica cartridge and purified by flash column chromatography (0–30% MeOH-DCM, containing 2% ammonium hydroxide). Fractions containing the desired product were pooled and concentrated to give the title (8.0 mg, 0.011 mmol, 22% yield) as a yellow solid. Accurate MS (ESI-QTof) calculated for $C_{41}H_{36}F_3N_8O_3$ $[M + H]^+$ 745.2862, found 745.2957. ^1H NMR (400 MHz, DMSO) δ 9.09 – 8.95 (m, 1H), 7.98 (dd, *J* = 9.0, 5.8 Hz, 1H), 7.52 – 7.32 (m, 7H), 7.26 (dd, *J* = 8.8, 4.9 Hz, 1H), 7.18 (d, *J* = 3.0 Hz, 1H). Non-aromatic protons were omitted due to the complexity of high-field NMR signals. ^{19}F NMR (376 MHz, DMSO) δ -110.75 (dd, *J* = 11.9, 6.6 Hz), -139.83 – -140.21 (m), -172.13 (dq, *J* = 59.0, 30.0 Hz).

Diazo-G12Di-3

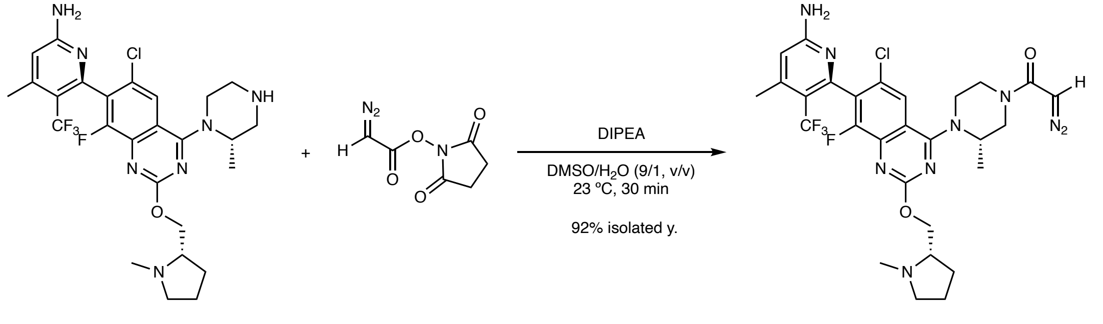

Diazo-G12Di-3

A 1-dram vial equipped with a stir bar was charged with (2,5-dioxopyrrolidin-1-yl) 2-diazoacetate (5.8 mg, 0.032 mmol) and 6-[6-chloro-8-fluoro-4-[rac-(2S)-2-methylpiperazin-1-yl]-2-[[rac-(2S)-1-methylpyrrolidin-2-yl]methoxy]quinazolin-7-yl]-4-methyl-5-(trifluoromethyl)pyridin-2-amine (Advanced ChemBlocks, 9.0 mg, 0.016 mmol). DMSO (0.45 mL), Water (0.05 mL) and N-ethyl-N-isopropyl-propan-2-amine (4.1 mg, 0.032 mmol) were added sequentially. The resulting homogeneous mixture was stirred at room temperature for 2 h, at which point full conversion of the limiting reagent was apparent. The reaction mixture was directly loaded onto a silica gel

cartridge and purified by flash column chromatography (0–40% MeOH-DCM, containing 2% ammonium hydroxide). Fractions containing the desired product were pooled and concentrated to give the title compound (9.3 mg, 0.015 mmol, 92% yield) as a white solid. Accurate MS (ESI-QTof) calculated for $C_{28}H_{31}ClF_4N_9O_2$ $[M + H]^+$ 636.2225, found 636.2264. ^1H NMR (400 MHz, DMSO) δ 7.80 (dd, *J* = 8.8, 1.5 Hz, 1H), 6.86 (s, 2H), 6.50 (s, 1H), 6.10 (s, 1H), 4.74 – 4.64 (m, 1H), 4.38 (ddd, *J* = 10.7, 8.7, 4.6 Hz, 1H), 4.23 – 4.04 (m, 2H), 3.62 (q, *J* = 13.1 Hz, 1H), 2.99 – 2.91 (m, 1H), 2.62 – 2.52 (m, 6H), 2.37 (d, *J* = 7.6 Hz, 6H), 2.18 (q, *J* = 8.5 Hz, 1H), 1.99 – 1.89 (m, 1H), 1.75 – 1.59 (m, 3H), 1.35 – 1.22 (m, 2H). ^{19}F NMR (376 MHz, DMSO) δ -53.60 (d, *J* = 3.5 Hz), -125.93 (d, *J* = 20.0 Hz).

Diazo-G12Di-4

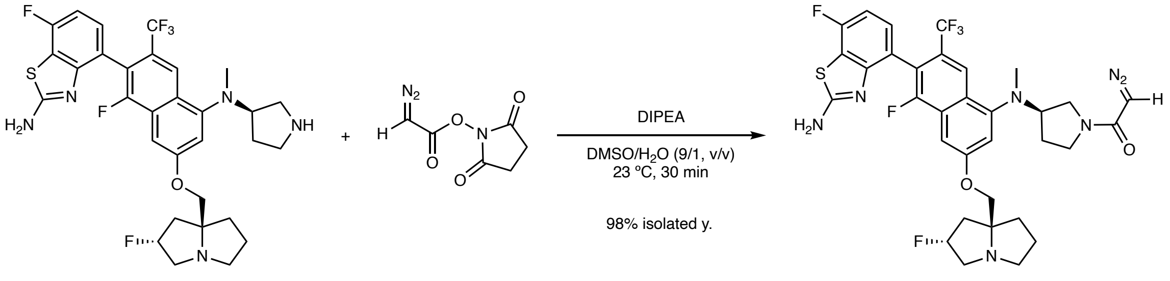

Diazo-G12Di-4

A 1-dram vial equipped with a stir bar was charged with (2,5-dioxopyrrolidin-1-yl) 2-diazoacetate (12.6 mg, 0.0689 mmol) and 7-fluoro-4-[8-fluoro-2-[[(2*R*,8*S*)-2-fluoro-1,2,3,5,6,7-hexahydropyrrolizin-8-yl]methoxy]-4-[methyl-[(3*R*)-pyrrolidin-3-yl]amino]-6-(trifluoromethyl)quinazolin-7-yl]-1,3-benzothiazol-2-amine (ChemScene, 22.0 mg, 0.0345 mmol). DMSO (0.45 mL) was added to dissolve the materials. Water (0.050 mL) and DIPEA (8.9 mg, 0.069 mmol) were added sequentially. The mixture was stirred at ambient temperature for 30 min when LC-MS indicated full consumption of the limiting reagent. The reaction mixture was directly loaded onto a silica cartridge and purified by RediSep Gold Silica Column (0–30% MeOH*-DCM, MeOH* was supplemented with 2% ammonium hydroxide). Fractions containing the desired product were pooled and concentrated to give the title compound as a white solid (23.8 mg, 0.0337 mmol, 98% isolated yield). Accurate MS calculated for $C_{31}H_{30}F_6N_9O_2S$ $[M + H]^+$ 706.2147, found 706.2249. ^1H NMR (400 MHz, DMSO) δ 8.24 (d, *J* = 2.9 Hz, 1H), 7.92 (s, 2H), 7.19 (ddd, *J* = 8.5, 5.6, 1.0 Hz, 1H), 7.04 (dd, *J* = 9.2, 8.4 Hz, 1H), 5.78 (s, 1H), 5.35 (s, 1H), 5.21 (t, *J* = 2.7 Hz, 1H), 5.15 (s, 1H), 4.15 (dd, *J* = 10.5, 2.5 Hz, 1H), 4.05 (dd, *J* = 10.4, 3.7 Hz, 1H), 3.34 (s, 3H), 3.17 – 3.06 (m, 2H), 3.02 (d, *J* = 2.2 Hz, 1H), 2.88 – 2.78 (m, 1H), 2.55 (s, 2H), 2.30 (s, 3H), 2.17 – 2.08 (m, 1H), 2.06 (d, *J* = 3.1 Hz, 1H), 2.00 (s, 1H), 1.88 – 1.75 (m, 4H). ^{19}F NMR (376 MHz, DMSO) δ -125.93 (d, *J* = 20.0 Hz).

Hydroxy-G12Di

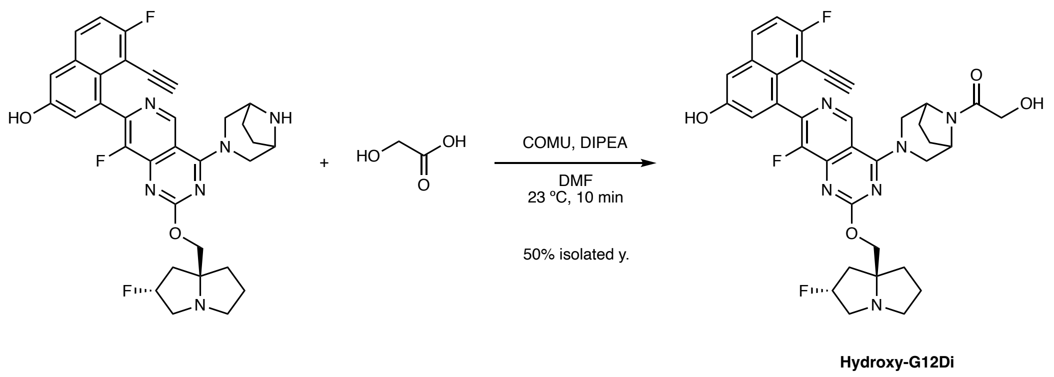

A 4-mL dram vial equipped with a stir bar was charged with MRTX1133 (20.0 mg, 0.0333 mmol), glycolic acid (3.0 mg, 0.040 mmol), and COMU (17.1 mg, 0.040 mmol). DMF (0.33 mL) and DIPEA (12.9 mg, 0.100 mmol) were added. The mixture was stirred at room temperature for 10 min before loaded onto a silica cartridge and purified by flash column chromatography using a 0–40% MeOH-DCM gradient. The title compound was obtained as a yellow solid in its hexafluorophosphate (1:1) form (13.4 mg,0.017 mmol, 50% yield). Accurate MS calculated for $C_{35}H_{34}F_3N_6O_4$ $[M + H]^+$ 659.2594, found 659.2585. ^{1}H NMR (400 MHz, DMSO) δ 10.72 (br, 1H), 10.20 (s, 1H), 9.10 (s, 1H), 8.00 (dd, *J* = 9.2, 5.9 Hz, 1H), 7.49 (d, *J* = 9.0 Hz, 1H), 7.47 – 7.39 (m, 1H), 7.19 (d, *J* = 2.6 Hz, 1H), 4.93 (t, *J* = 5.8 Hz, 1H), 4.72 (s, 1H), 4.50 (s, 1H), 4.47 – 4.40 (m, 2H), 4.19 (d, *J* = 4.9 Hz, 2H), 3.93 (d, *J* = 5.3 Hz, 1H), 3.78 (t, *J* = 13.8 Hz, 1H), 3.67 – 3.53 (m, 3H), 3.15 (qd, *J* = 7.3, 4.1 Hz, 2H), 2.42 (s, 1H), 2.35 (s, 1H), 2.28 – 2.19 (m, 1H), 2.07 (s, 1H), 1.95 (s, 1H), 1.78 (s, 1H), 1.30 – 1.17 (m, 5H). ^{19}F NMR (376 MHz, DMSO) δ -110.63 (t, *J* = 7.9 Hz), -139.82 (dd, *J* = 68.7, 11.1 Hz), -172.8 (m).

Covalent Docking

Covalent Docking Workflow

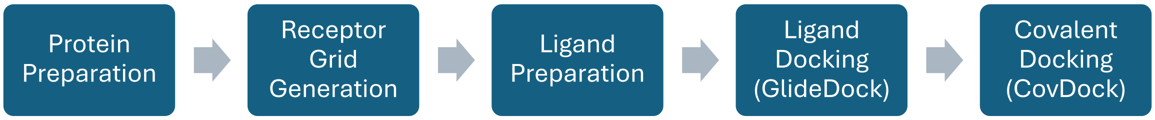

Protein Preparation

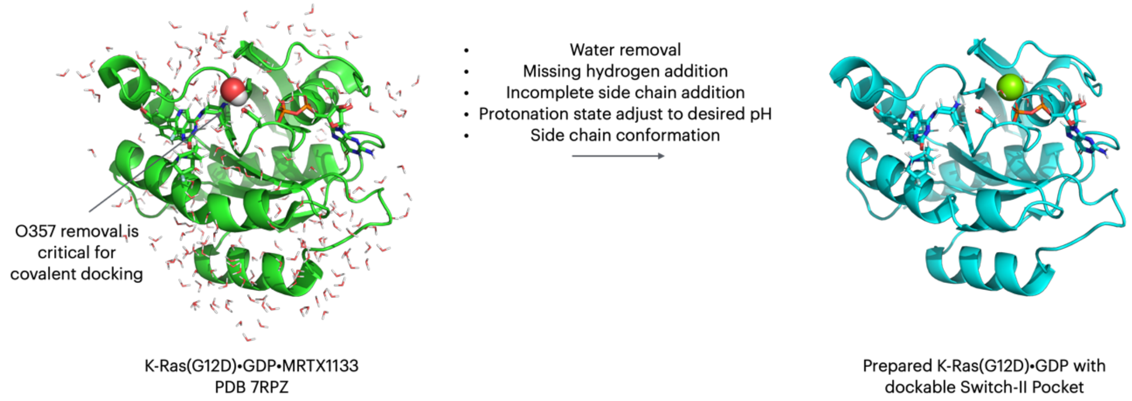

Receptor Grid Generation

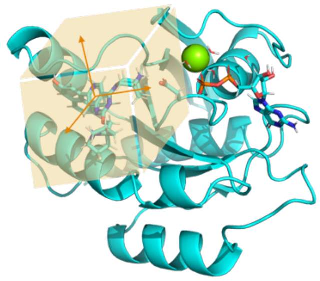

20 Å cubic grid centered at the MRTX1133 ligand
Proposed ligand will be docked within this grid

Ligand Preparation

Docked compounds and respective SMILES strings are listed in the table,

Compound	SMILES
**Diazo-G12Di-1**	F[C@@H](C1)C[C@]2(N1CCC2)COC(N=C3N4C[C@H]5N(C([C-]([H])[N+]#N)=O)[C@H](CC5)C4)=NC6=C3C=NC(C7=C(C(C#C)=C(F)C=C8)C8=CC(O)=C7)=C6F
**Diazo-G12Di-1-Proxy** (α-chloroacetamide instead of α-diazoacetamide)	F[C@@H](C1)C[C@]2(N1CCC2)COC(N=C3N4C[C@H]5N(C(CCl)=O)[C@H](CC5)C4)=NC6=C3C=NC(C7=C(C(C#C)=C(F)C=C8)C8=CC(O)=C7)=C6F

The ligand preparation process was exemplified by **Diazo-G12Di-1**.

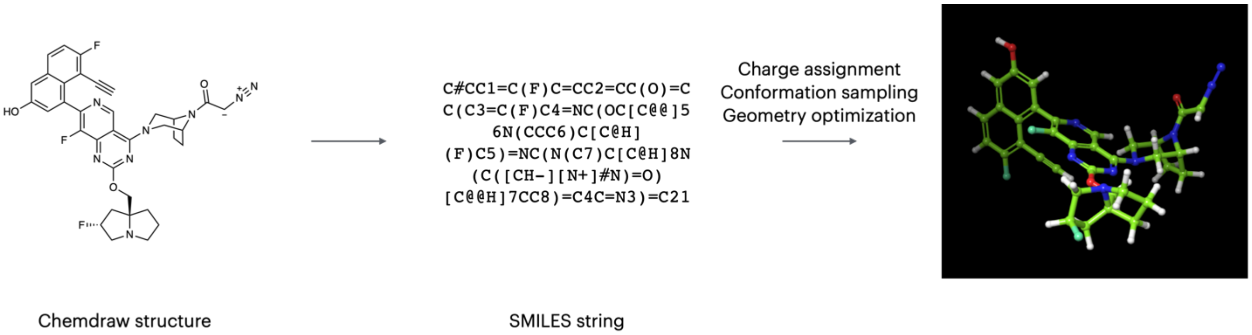

Ligand Docking

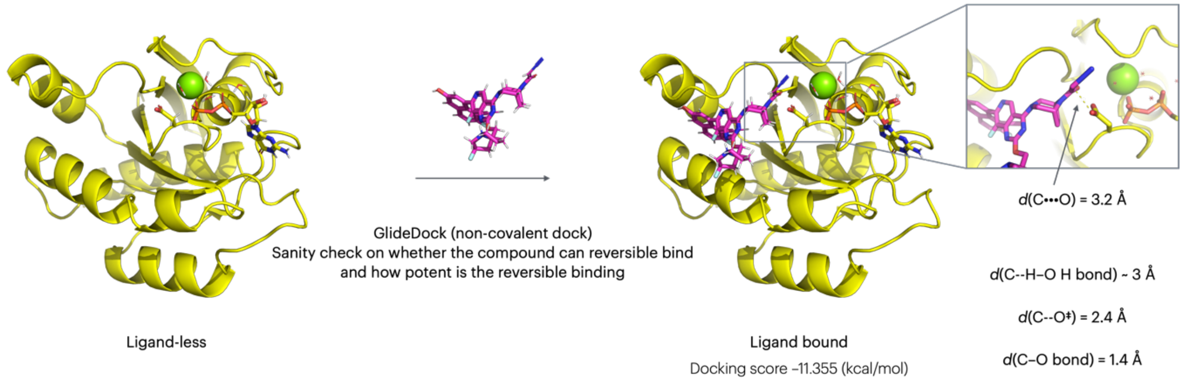

Ligand Docking suggested that **Diazo-G12Di-1**, the diazoacetamide of MRTX1133, was able to bind K-Ras(G12D). The docked structure also suggested an optimal conformation for the covalent reaction. The electrophilic and nucleophilic atom pairs (C•••O) had a distance and angle right for bond formation.

Covalent Docking

Because CovDock concerns only about the starting receptor-ligand energy and final complex and does not take chemical reaction energy change into consideration, we designed a proxy molecule that have an α-chloroacetamide instead of α-diazoacetamide for CovDock for simplicity of CovDock file.

The customized chemical reaction type was defined as following:

```
# QZ defined reaction: Nucleophilic Substitution Nucleophilic
substitution of a ligand halide by a receptor carboxylic acid.
#
# Receptor ASP/GLU, oxygen is <1>
RECEPTOR_SMARTS_PATTERN 3,[C](=[O])-[O-]

# Ligand, atom connected to halide is <2>
```

```
LIGAND_SMARTS_PATTERN 1,[*][Cl]

# Neutralize the ASP/GLU, if necessary
CUSTOM_CHEMISTRY ("<1>",("charge",0,(1)))

# Add ligand-receptor single bond
CUSTOM_CHEMISTRY ("<1>|<2>",("bond",1,(1,2)))

# Remove the halogen leaving group
CUSTOM_CHEMISTRY ("<2>[Cl]",("delete",2))
```

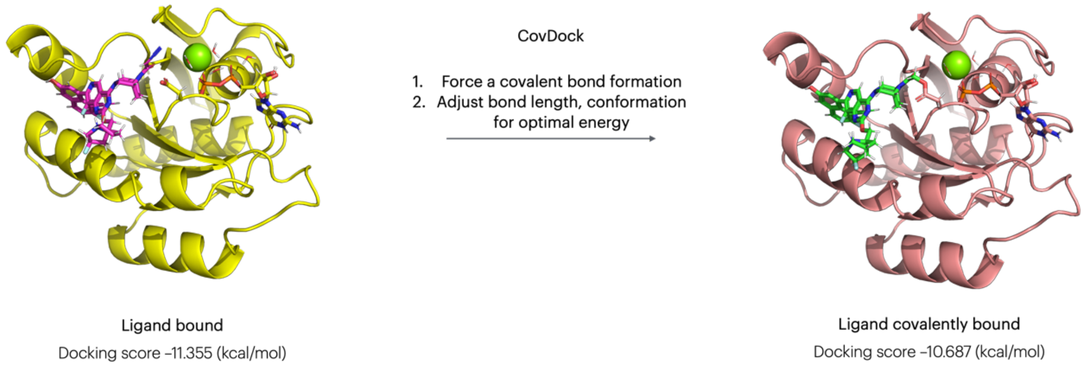

Note: The two docking scores have not taken the reaction ΔH into consideration. Therefore, it should not be concluded that the reaction was not spontaneous.

Reference

1 Zheng, Q., Zhang, Z., Guiley, K. Z. & Shokat, K. M. Strain-release alkylation of Asp12 enables mutant selective targeting of K-Ras-G12D. *Nat Chem Biol* **20**, 1114-1122 (2024).

2 Richter, M. J. R., Zecri, F. J., Briner, K. & Schreiber, S. L. Modular Synthesis of Cyclopropane-Fused N-Heterocycles Enabled by Underexplored Diazo Reagents. *Angew Chem Int Ed Engl* **61**, e202203221 (2022).

3 Jun, J. V. & Raines, R. T. Two-Step Synthesis of alpha-Aryl-alpha-diazoamides as Modular Bioreversible Labels. *Org Lett* **23**, 3110-3114 (2021).

4 Jun, J. V., Petri, Y. D., Erickson, L. W. & Raines, R. T. Modular Diazo Compound for the Bioreversible Late-Stage Modification of Proteins. *J Am Chem Soc* **145**, 6615-6621 (2023).

5 Bew, S. P., Ashford, P. A. & Bachera, D. U. Synthesis of Structure and Function Diverse α-D-Diazoacetates, α-D-Diazoacetamides, α-D-Diazoketones, and the Antibiotic α-D-Azaserine. *Synthesis-Stuttgart* **45**, 903-912 (2013).

NMR Spectra

<u>Diazo-G12Di-1</u>, ^{1}H NMR (DMSO-d6, 400 MHz)

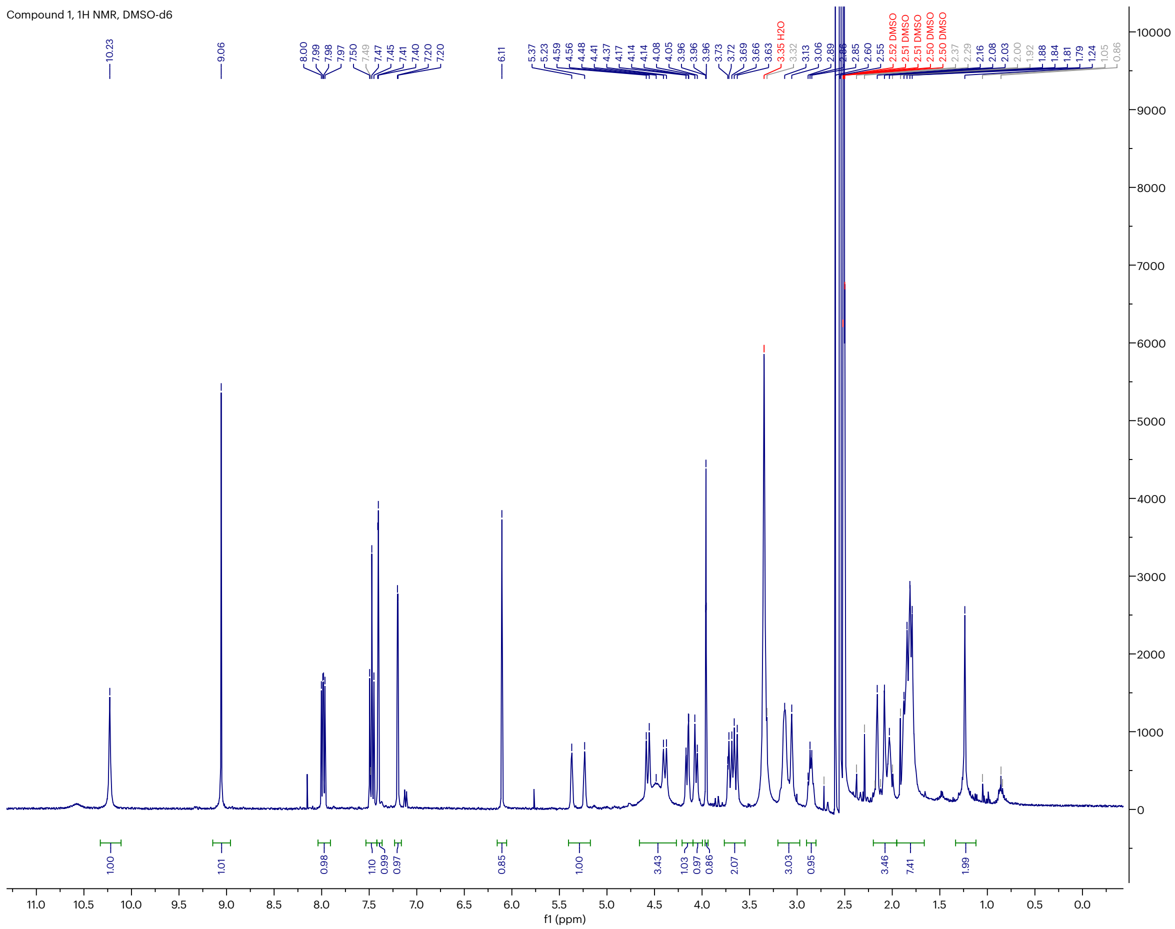

Diazo-G12Di-1, ^{19}F NMR (DMSO-d6, 376 MHz)

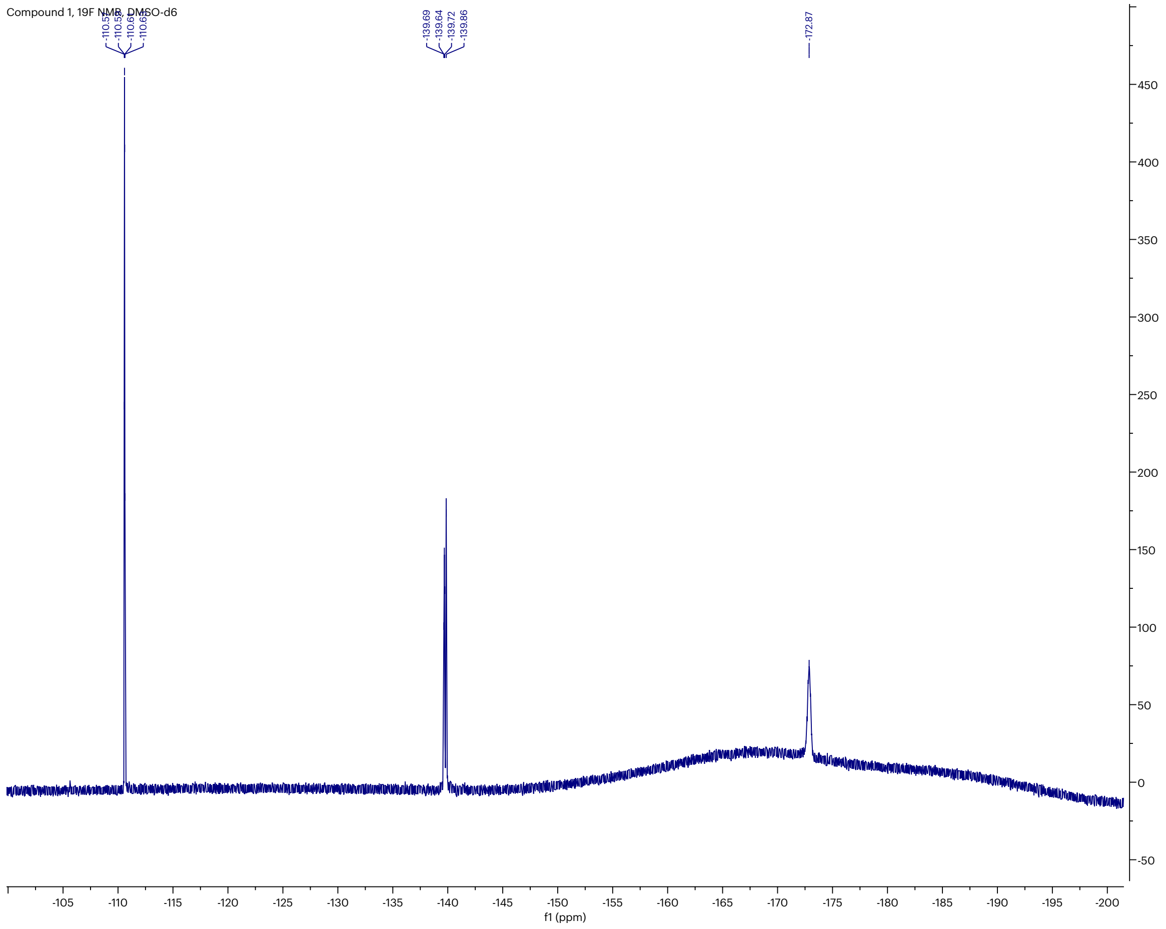

Diazo-G12Di-2, ^{1}H NMR (DMSO-d6, 400 MHz)

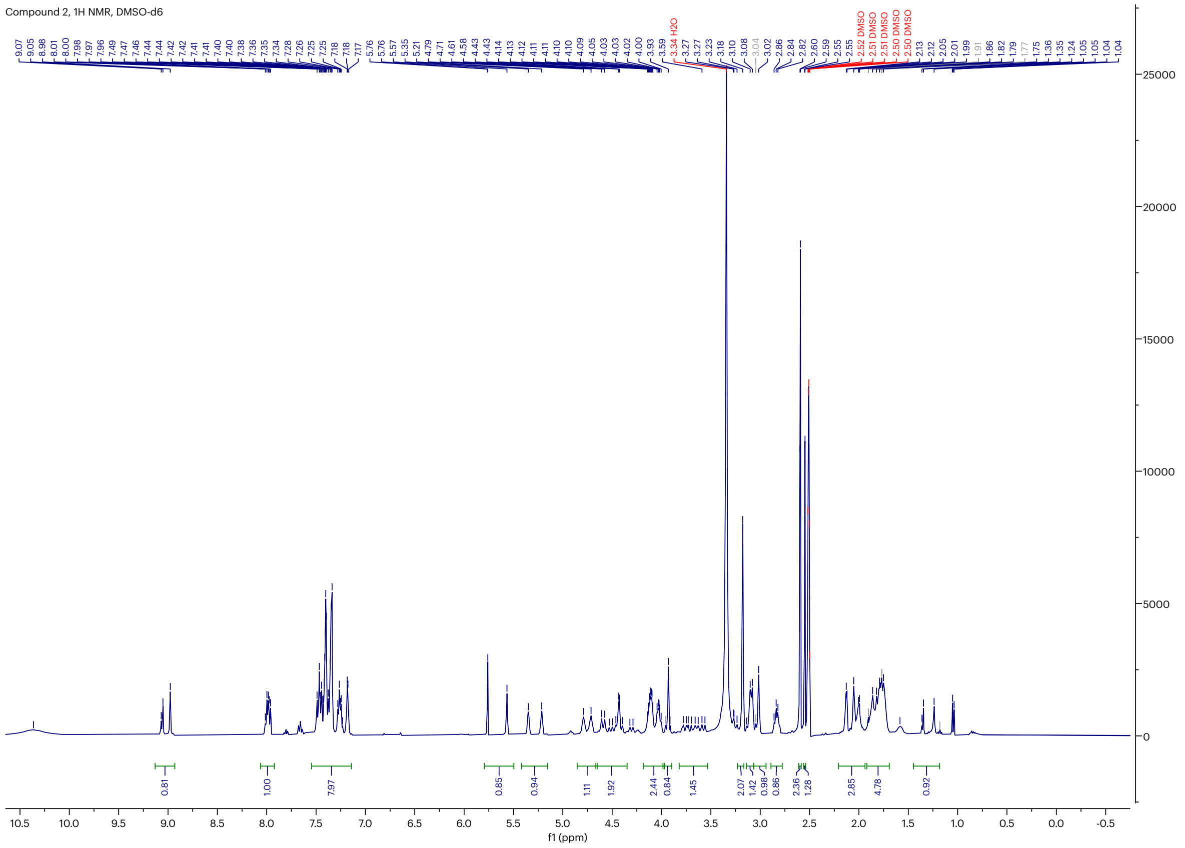

Diazo-G12Di-2, ^{19}F NMR (DMSO-d6, 376 MHz)

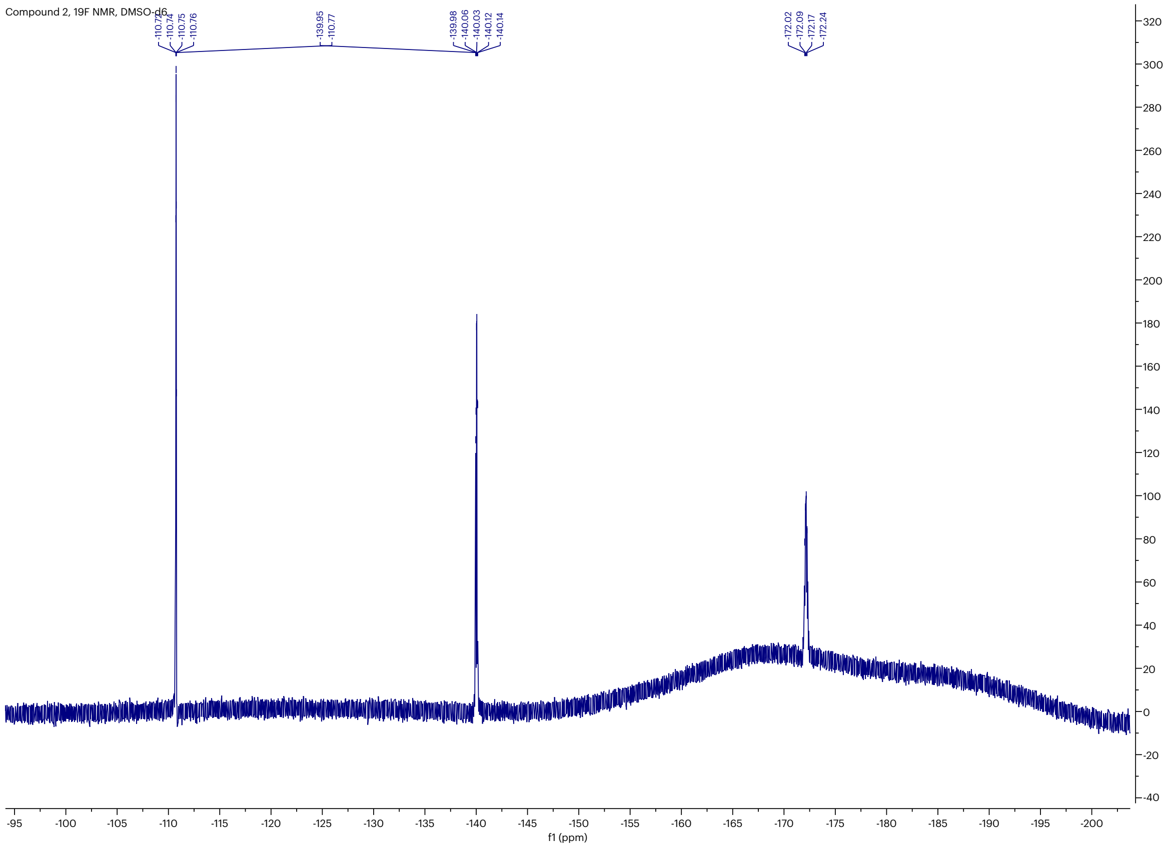

Diazo-G12Di-3, ^{1}H NMR (DMSO-d6, 400 MHz)

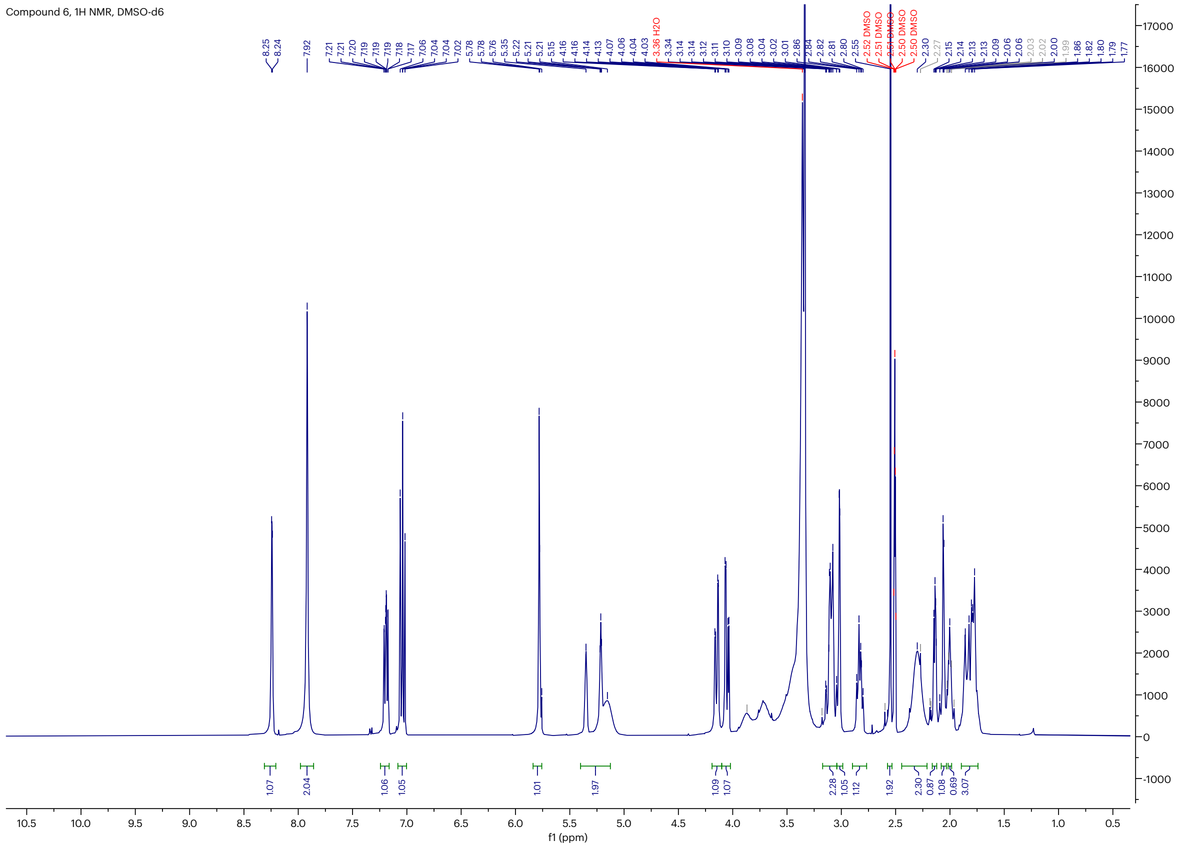

Diazo-G12Di-3, ^{19}F NMR (DMSO-d6, 376 MHz)

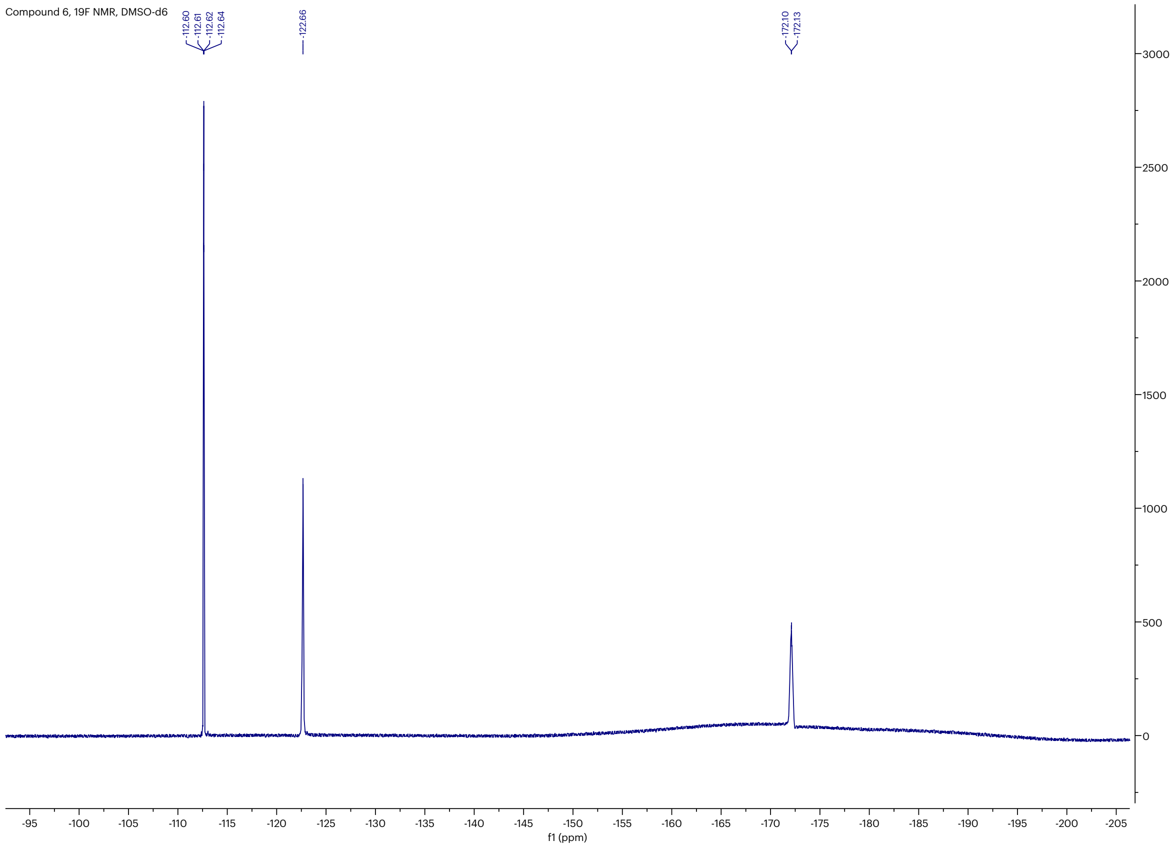

Diazo-G12Di-4, ^{1}H NMR (DMSO-d6, 400 MHz)

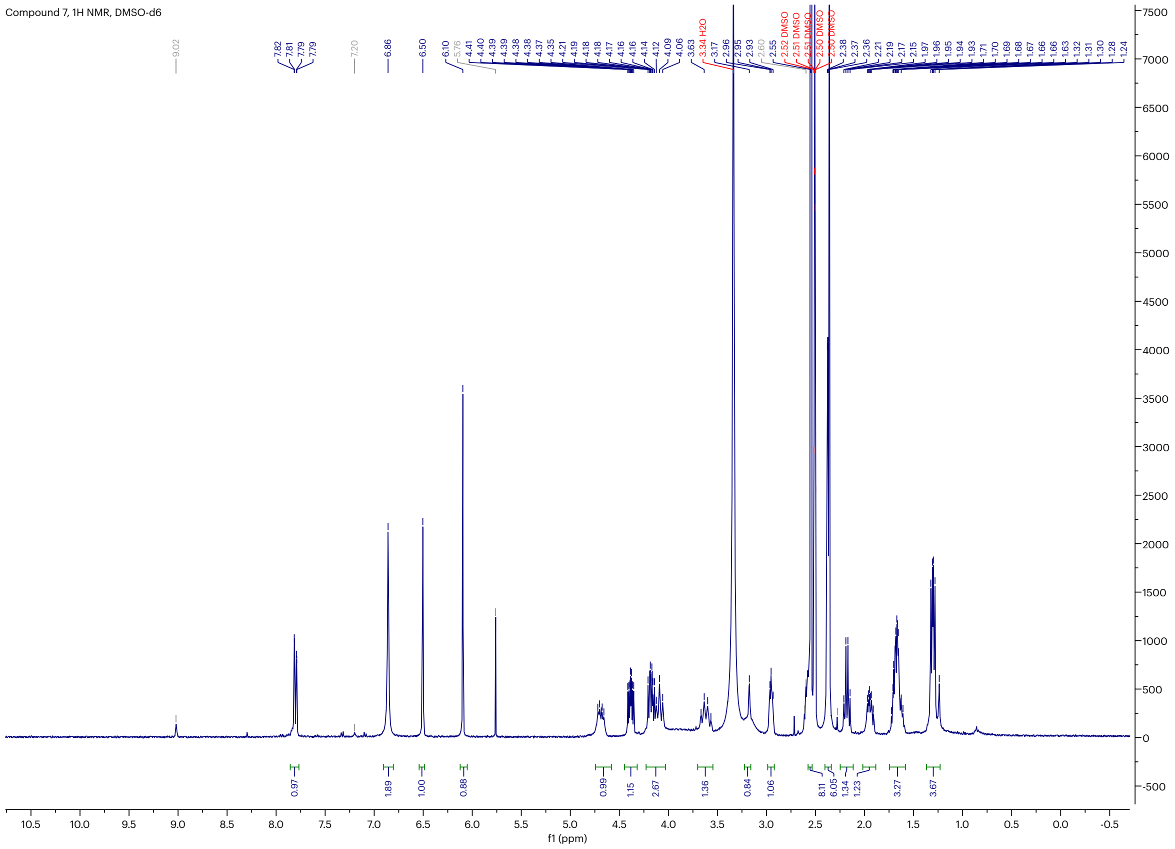

<u>Diazo-G12Di-4</u>, <u>^{19}F NMR (DMSO-d6, 376 MHz)</u>

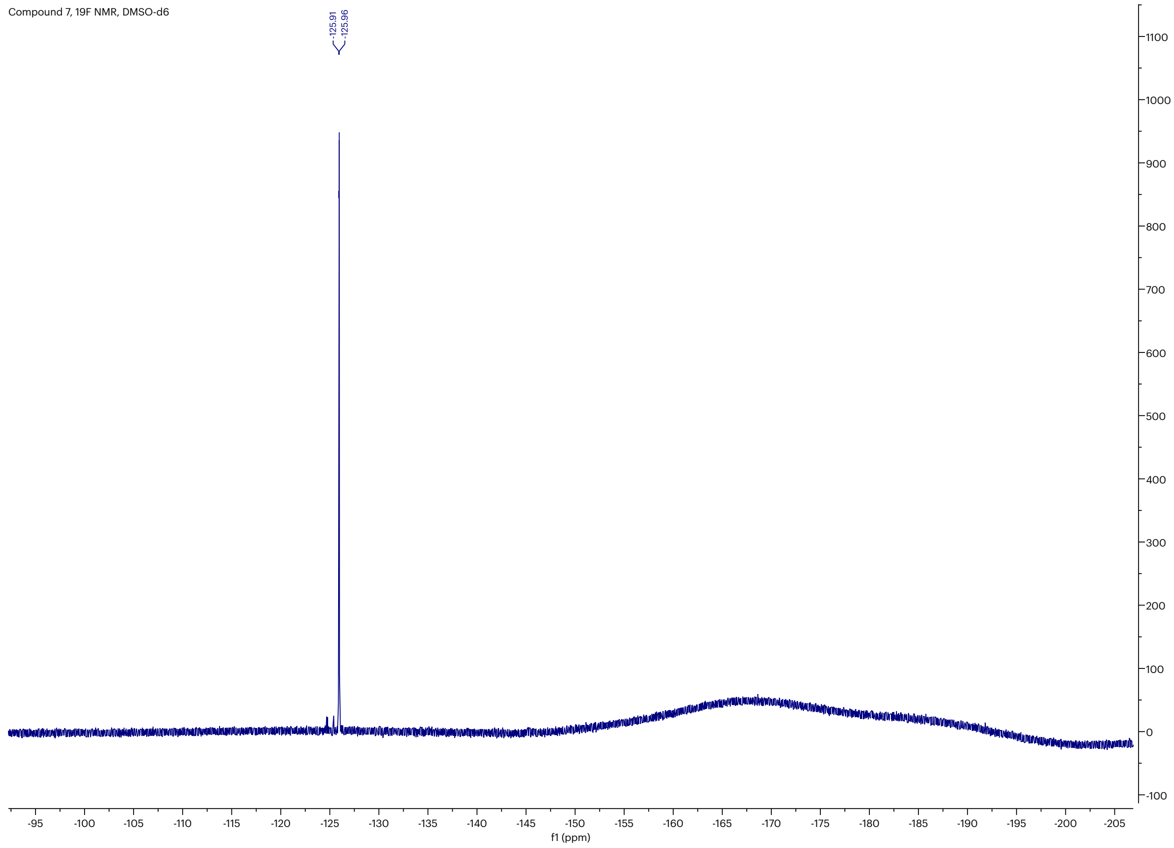

Hydroxy-G12Di, ^{1}H NMR (DMSO-d6, 400 MHz)

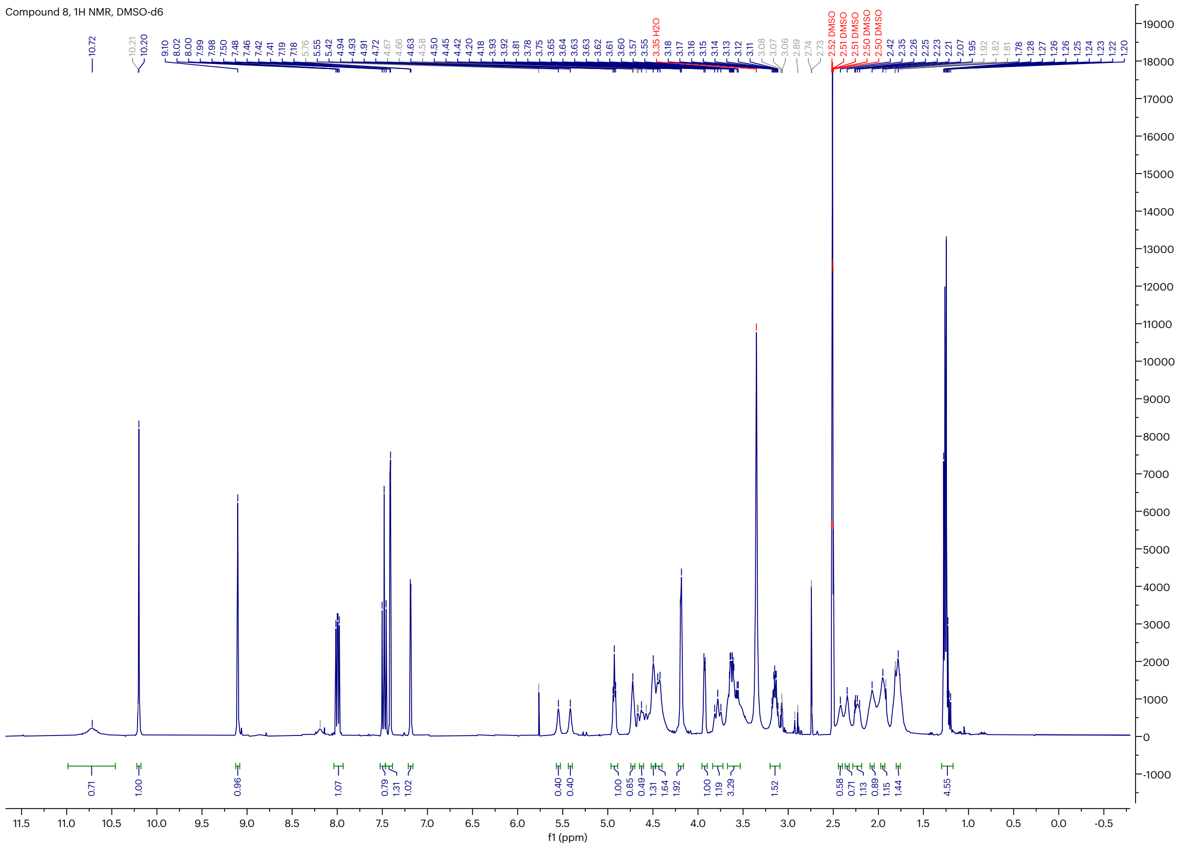

Hydroxy-G12Di, ^{19}F NMR (DMSO-d6, 376 MHz)

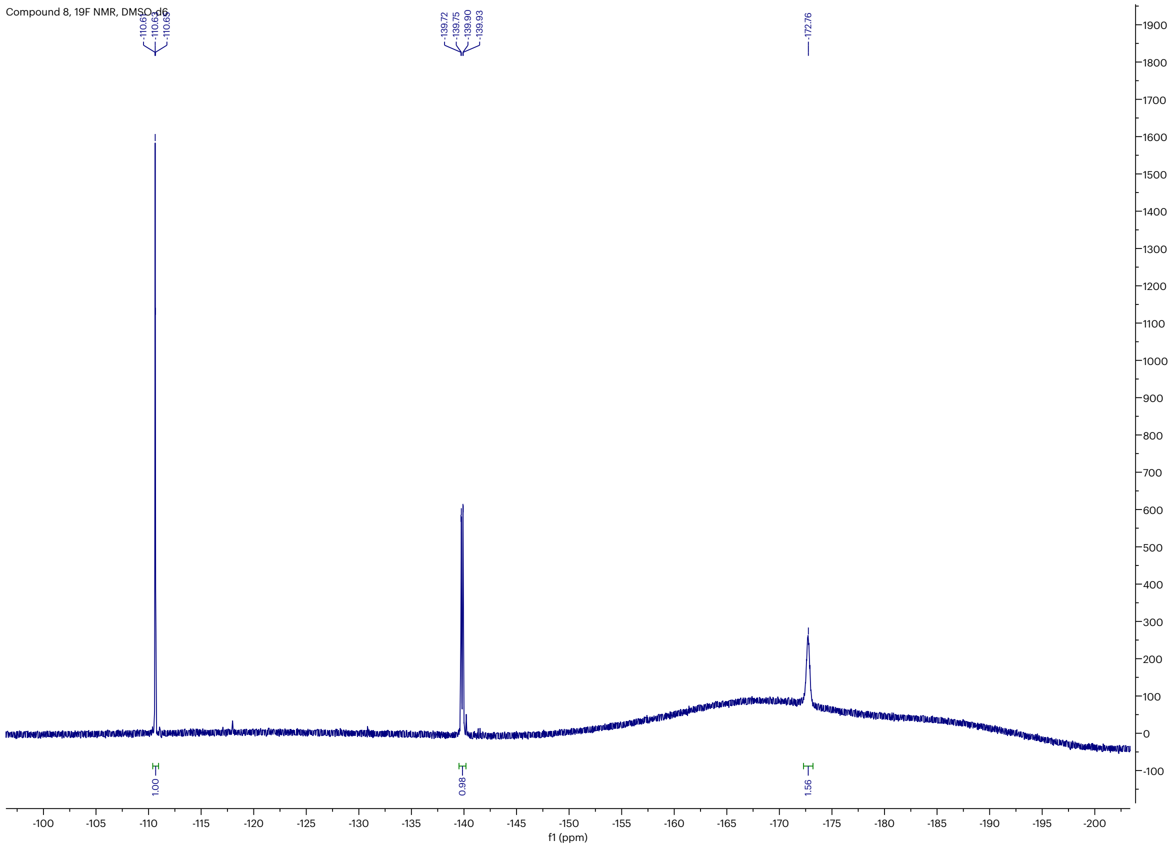

Supplement: Supplementary file 1 [file ja5c06745_si_001.pdf]
